# Diversity and spatiotemporal dynamics of fish communities in the Chongqing section of the upper Yangtze River based on eDNA metabarcoding

**DOI:** 10.1002/ece3.10681

**Published:** 2023-11-10

**Authors:** Yanjun Shen, Yufeng Zhang, Ruli Cheng, Wei Wang, Cong Duan, Zhihao Liu, Qiliang Chen, Yingwen Li, Meng Wang, Yang Luo

**Affiliations:** ^1^ Laboratory of Water Ecological Health and Environmental Safety, School of Life Sciences Chongqing Normal University Chongqing China; ^2^ Chongqing Rare and Endemic Fish National Nature Reserve Management Office Chongqing China

**Keywords:** biodiversity, environmental DNA, fish detection, the upper Yangtze River

## Abstract

Fish diversity plays a critical role in maintaining the balance of water ecosystems, especially in the Chongqing section of the National Nature Reserve for Rare and Endemic Fishes in the upper Yangtze River, which serves as an important habitat for rare and endemic fish, as well as an important channel for the replenishment of fishery resources in the Three Gorges Reservoir. Under a 10‐year ban on fishing in the Yangtze River basin, we investigate fish diversity and seasonal variation in the Reserve by using environmental DNA (eDNA) metabarcoding. We found fishes belonging to 85 genera, 24 families, and 8 orders in the Reserve. A comparison of eDNA metabarcoding results with the diversity of a recent fish catch revealed that eDNA metabarcoding not only enables rapid and efficient fish monitoring but also has a high sensitivity. Furthermore, the study demonstrates that eDNA metabarcoding can be used as a tool for monitoring seasonal variations of fish composition in freshwater ecosystems. The alpha and beta diversity analysis both showed compositional differences in the fish community in accordance with seasonal variations. In addition, changes in eDNA relative sequence abundance and the detection of fish species at different sampling sites may reflect shifts in habitat use and distribution. Thus, we provide detailed seasonal data on fish diversity in the Chongqing section of the Reserve. This will contribute to conservation and to the understanding of fish diversity and community dynamics in the Chongqing section of the Reserve.

## Graphical abstract

The upper Yangtze River is of great significance to maintaining freshwater fish germplasm resources and fish diversity in China. Under a 10‐year ban on fishing in the Yangtze River basin, eDNA metabarcoding has enormous advantages in freshwater biodiversity biomonitoring. Here we investigated fish diversity and seasonal variation in the Chongqing section of the National Nature Reserve for Rare and Endemic Fishes in the upper reaches of the Yangtze River by using eDNA metabarcoding. Based on eDNA metabarcoding, the Reserve had 132 species of fish belonging to 85 genera, 24 families, and 8 orders. A comparison of eDNA metabarcoding results with the diversity of a recent fish catch revealed that eDNA metabarcoding not only enables rapid and efficient fish monitoring but also has a high sensitivity. Furthermore, the study demonstrates that eDNA metabarcoding can be used as a tool for monitoring seasonal variations of fish composition in freshwater ecosystems. The alpha and beta diversity analyses both showed compositional differences in the fish community in accordance with seasonal variations. In addition, changes in eDNA relative sequence abundance and the detection of fish species at different sampling sites may reflect shifts in habitat use and distribution. Thus, we provide detailed seasonal data on fish diversity in the Chongqing section of the Reserve. This will contribute conservation and understanding of the fish diversity and community dynamics in the Chongqing section of the Reserve.

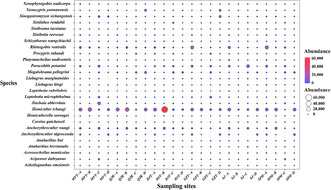

## 1 INTRODUCTION

The diversity of fish species is of utmost importance in the preservation of water ecosystems and the promotion of socially sustainable development (Liu & Dai, 2018; Magurran et al., 2011; Margules & Pressey, 2000). Situated in a topographically diverse region with ample precipitation and varying hydrological conditions, the Chongqing section of the National Nature Reserve for Rare and Endemic Fishes in the upper Yangtze River, referred to as “the Reserve” for conciseness, spans a length of 118.8 km and has been instrumental in fostering a bountiful fish population. The Reserve serves as a crucial habitat for both rare and endemic fish species, playing a vital role in replenishing fishery resources in the Three Gorges Reservoir (Qin et al., 2008). However, in recent years, a multitude of factors including habitat destruction, overfishing, climate change, pollution, and the invasion of alien species have led to a significant decline in fishery resources in the Reserve. This decline is particularly evident in the sharp decrease and depletion of populations of numerous rare and endemic fish species (Esguícero & Arcifa, 2010; Fahrig, 2002; Gangloff et al., 2016; Gao et al., 2013; Wei, 2012; Xiong et al., 2014; Yang et al., 2014). In order to safeguard the biodiversity and natural habitats of rare and indigenous fish species, a decade‐long prohibition on fishing was enforced in the Yangtze River Basin in 2020 (Cao, 2022). This extensive measure encompasses a complete cessation of all fishing activities in the basin, with the objective of facilitating the recuperation of fish populations and guaranteeing the enduring viability of fishery resources.

The successful evaluation of fishery resources recovery and the monitoring of fishery resource diversity necessitates the implementation of continuous and protracted surveys (Wang et al., 2020). Traditional fish catch survey methods have certain limitations in terms of comprehensively detecting biodiversity. These limitations arise from low sampling efficiencies, potential harm to organisms, and the need for taxonomic expertise (Causey et al., 2004; Olds et al., 2016; Trimble & Van Aarde, 2012; Zhu et al., 2021). Moreover, conducting large‐scale traditional fish resource surveys becomes challenging in the framework of the 10‐year fishing ban in the Yangtze River Basin. Hence, it is imperative to employ an efficient and dependable fish diversity monitoring technique that is not influenced by specific ecosystems, in order to effectively track the alterations and recuperation impacts of environmental enhancements on fish diversity (Sigsgaard et al., 2017).

The utilization of molecular methodologies has significantly enhanced biodiversity evaluation in numerous ecosystems and regions that are abundant in biodiversity (Schwartz et al., 2006). Among these molecular approaches, the analysis of environmental DNA (eDNA), which involves the examination of genetic material discharged into the environment (e.g., water, air, soil, and sediment) by organisms through their skin, saliva, and secretions, has proven to be particularly valuable. In recent years, eDNA metabarcoding has emerged as a highly sensitive method for species detection and biodiversity assessment, offering significant advantages over traditional fish‐catching methods in terms of minimizing ecosystem disturbance (Bylemans et al., 2018; Doi et al., 2021; Fujii et al., 2019; Laramie et al., 2015; Thomsen & Willerslev, 2015). Consequently, it has been extensively and successfully employed in studies investigating fish diversity in various aquatic ecosystems, including the coast of Japan (Yamamoto et al., 2017), the Yangtze River (Xu & Chang, 2016; Qu et al., 2020; Wang et al., 2022; Cheng, Luo, Li, Zhang, Liu, et al., 2023), and the Wujiang River (Cheng, Luo, Zhang, Li, Li, & Shen, 2023; Cheng, Luo, Zhang, Li, Wang, et al., 2023). eDNA metabarcoding has the potential to enhance the comprehension of assemblage compositions and streamline the evaluation of freshwater fish diversity in comparison to conventional sampling methods (Cilleros et al., 2018). This advantage primarily stems from the heightened sensitivity of eDNA metabarcoding in detecting the existence of uncommon and elusive fish species. By directly capturing genetic material from the environment, eDNA metabarcoding enables the identification of species even when they occur in low quantities or are challenging to observe using traditional approaches (Jerde et al., 2013; Valentini et al., 2016). Moreover, the utilization of eDNA metabarcoding facilitates the concurrent identification of numerous species in a solitary sample, thereby enabling a more extensive evaluation of species diversity and community structure. This methodology effectively surmounts the constraints imposed by conventional techniques, which frequently suffer from limitations in terms of sampling intensity, time consumption, and taxonomic proficiency. Consequently, the capacity of eDNA metabarcoding to offer comprehensive and expedient assessments of species has rendered it a crucial instrument in the realm of biodiversity research and conservation endeavors (Hinlo et al., 2017).

In order to evaluate the fish species composition and biodiversity in the Reserve, a comprehensive and systematic monitoring approach was employed using eDNA metabarcoding of water samples collected from six designated sites throughout the four seasons spanning the period from 2021 to 2022. Additionally, the objective of this study was to analyze the spatial and temporal distribution patterns of fish populations, as well as to assess the impacts of the recently implemented fishing ban in the Yangtze River Basin. By doing so, this research aims to contribute to the knowledge and conservation efforts surrounding fish diversity.

## 2 METHODS

### 2.1 Study area

**FIGURE 1:**
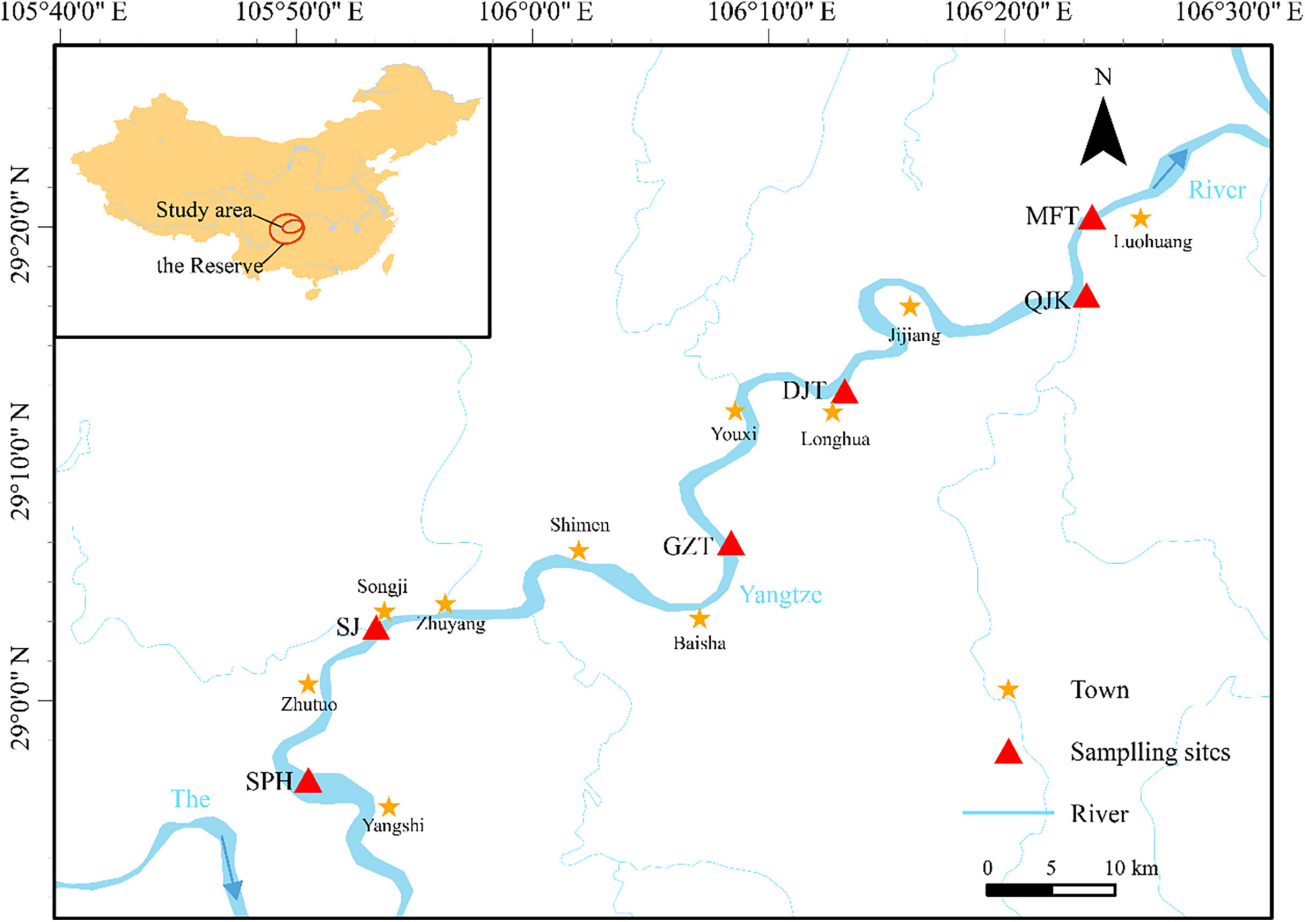
The location of sampling sites in the Reserve. ArcGIS was used for mapping.

We collected water samples in summer (June 2021), autumn (October 2021), winter (January 2022), and spring (March 2022). In order to obtain eDNA samples with high initial concentrations, six sampling sites were established based on the fish spawning grounds in the Reserve (Figure 1), namely Sanpao River (SPH), Songji (SJ), Gaozhantan (GZT), Dingjiatuo (DJT), Qijiangkou (QJK), and Mafutuo (MFT) (He et al., 2018). Among these sites, the SPH site is situated in the core area of the Reserve, near the Zhaojia Dam in the Nihao section, where the water flow exhibits turbulence characterized by rapids and eddies. The SJ site is situated at the interface between the core area and the buffer zone, in close proximity to Songji Ancient Town. This location experiences the accumulation of sand and stones in the stone beam that extends towards the river, as well as the presence of multiple water eddies. The GZT, DJT, and MFT sites are located in the experimental section of the Reserve, where there is less human interference, mainly natural river channels. The QJK site is positioned at the confluence of the tributary (Qijiang) and the main stream (Yangtze River). This site exhibits intricate topography, sedimentary bottom, dense coastal vegetation, and numerous depressions, leading to a significant level of habitat heterogeneity.

### 2.2 eDNA sampling and extraction

This investigation was carried out with the approval of relevant fishery authorities, taking into account the 10‐year ban on fishing in the Yangtze River basin. A plexiglass material water collector was used to collect a total of 6 L of water from the left, middle, and right sections of each sampling site. Subsequently, the collected water samples were mixed and divided into three disinfected polyethylene bottles, creating three replicate samples (Janosik & Johnston, 2015; Luo, 2019; Xu & Chang, 2016). In order to prevent contamination, the sampling equipment underwent sterilization using a 10% solution of commercial bleach, while the collection personnel utilized fresh disposable gloves for each step of the sampling procedure (Gelis et al., 2021; Pilliod et al., 2013). Within a time frame of 6 hours, the eDNA present in water samples was collected onto 0.45 μm nitrocellulose filter membranes (Whatman, UK) with the assistance of a peristaltic pump (Auto‐Science, Tianjin, China). Prior to and following the suction filtration of each sample, the equipment underwent sterilization to eliminate any remaining DNA and prevent cross‐contamination between samples (Goldberg, 2016; Shu et al., 2020). Furthermore, as part of the sampling process at each site, a negative control was implemented by filtering 2 L of distilled water to assess the presence of exogenous DNA contamination. Subsequently, the filter membranes were promptly transferred into sterile 5‐mL plastic scintillation tubes, rapidly frozen in liquid nitrogen, and preserved at −80°C until DNA extraction.

The extraction of total DNA from water samples was conducted using PowerWater DNA Isolation Kits (Qiagen) by extracting it from the filtration membrane. In order to minimize DNA degradation, a sterile Tris‐ethylenediaminetetraacetic acid solution was utilized for the final elution step instead of PW6 (sterile eluent). Subsequently, the quality of the DNA was assessed through 1% agarose gel electrophoresis. Each sample was extracted independently, with a negative control in the form of a blank filter membrane set up in parallel. By comparing the results of the samples with those of the blank membrane, any contamination that may have occurred during the experimental process was evaluated (Goldberg, 2016; Shu et al., 2020; Wang et al., 2022). Subsequently, the DNA samples were stored at −20°C pending PCR amplification.

### 2.3 DNA amplification and sequencing

The eDNA samples were subjected to amplification using the fish universal primers Tele02‐F (5′‐ AAACTCGTGCCAGCCACC‐3′) and Tele02‐R (5′‐GGGTATCTAATCCCAGTTTG‐3′), which specifically target the 12S rRNA region of the mitochondrial genome for the purpose of identifying fish species (Taberlet et al., 2018). The amplification process was carried out in a 20 μL reaction system, consisting of 4 μL 5 × FastPfu Buffer, 2 μL deoxynucleoside triphosphates, 0.4 μL FastPfu Polymerase, 2–5 μL template DNA, and 0.8 μL forward and reverse primers. The reaction system was then supplemented with ddH2O to reach a final volume of 20 μL. The PCR reaction was conducted in the following manner: an initial denaturation step at 95°C for 5 min, followed by 27 cycles of denaturation at 95°C for 30 s, annealing at 55°C for 30 s, extension at 72°C for 45 s, and a final extension step at 72°C for 10 min. Three replicates of the PCR were performed for each sample, then replicate PCR products were combined. The verification of PCR products was accomplished through 2% agarose gel electrophoresis. All samples yielded detectable PCR products, while none of the negative controls exhibited any products. The PCR products were then purified using the AxyPrep DNA Gel Extraction Kit and eluted with Tris–HCl. Finally, the Illumina PE250 library preparation was established, and high‐throughput sequencing was performed using the Illumina NovaSeq 6000 sequencing platform, conducted by Biozeron (Shanghai, China).

### 2.4 Bioinformatics and statistical analyses

In the initial step, Trimmomatic v.0.36 (Bolger et al., 2014) was employed to eliminate poor‐quality sequences (Magoc & Salzberg, 2011). This involved filtering out bases with a read tail quality value below 20, implementing a window size of 10 bp, truncating back‐end bases from the window if the average quality value within the window fell below 20, and discarding reads shorter than 100 bp. Additionally, the remaining paired reads were merged into a single sequence using FLASH (v.1.2.11). Subsequently, Usearch (version 10, http://drive5.com/uparse/) software was utilized to identify and eliminate chimeric sequences. The process entailed conducting a comparison between the sequences and the reference sequences sourced from the GOLD database, alongside de novo sequences. The removal of primers was done with Cutadapt (v4.0) (Martin, 2011). Usearch was employed to process high‐quality sequences, generating “parent–child” sets exhibiting 97% similarity (Zhang et al., 2022), thereby obtaining unique sequences. The subsequent stage encompassed a comparison and annotation of the unique sequences against the NCBI public database, employing the Blastn tool and the uclust algorithm. During this procedure, a preliminary taxonomic annotation table, known as the molecular operational taxonomic unit (MOTU) annotation table, was produced. The creation of MOTUs was carried out with Usearch, with a similarity threshold of ≥97%, an e‐value of ≤10–5, and a coverage of ≥0.9 (Edgar, 2010). The annotations acquired from the public database underwent manual filtration to eliminate MOTU sequences associated with non‐fish organisms. Additionally, we analyzed traditional catch survey data from the Reserve over the past decade based on some references (Gao et al., 2013; He et al., 2018; Tian et al., 2016) to exclude MOTU sequences that were improbable to pertain to the region. The 12S rRNA and mitochondrial genome sequences of all freshwater fish species were collected from the NCBI nucleotide database (https://www.ncbi.nlm.nih.gov/) and employed as a reference database for eDNA annotation. Finally, MOTUs with less than 10 reads were excluded (Shu et al., 2021; Zhang et al., 2022). To ensure comparability across samples, the reads from each sample were randomly selected and normalized using QIIME v.1.9.0, based on the minimum number of sequences obtained in any of the samples (Caporaso et al., 2010). This normalization procedure maintained a consistent relative sequence abundance (read ratio) for each species in all samples. The average values of the three replicate samples were computed and utilized for subsequent analyses, encompassing species composition, alpha diversity, and beta diversity. The traditional catch survey data includes 103 freshwater and 8 alien species fish species belonging to 69 genera, 19 families, and 7 orders (Table A1). Among them are 4 species of state‐level protected fish, 22 species of endemic fish, and 5 species of Chongqing municipal key protected fishes.

The calculation of the relative abundances of fish species was conducted using the MOTU abundance tables and species count tables at the species taxonomic level. The species composition of fish was revealed through the creation of bar charts, which were based on the detected relative sequence abundances of species. Furthermore, the relationship among the sampling sites was analyzed using the unweighted pair group mean average (UPGMA), taking into account the similarity degree of fish species among each sample (Figure 6). Diversity indices, namely Shannon, Pielou, Simpson, and Richness, were computed with the “vegan” package in R for the purpose of examining and contrasting fish diversity across various sampling sites (Oksanen et al., 2007). The Richness index serves as an indicator of species richness, while the Pielou index provides insights into the evenness of species in the community (Chen, 2020). The Simpson and Shannon indices are widely employed metrics for assessing community diversity, with the former exhibiting a negative correlation with diversity (Shannon, 1948; Simpson, 1949). Furthermore, in order to elucidate temporal and spatial variations in the structure of fish communities, Principal Coordinates Analysis (PCoA) was conducted using the Bray‐Curtis matrix (Jurasinski & Retzer, 2012). Kruskal‐Wallis test and One‐way ANOVA analysis were performed to test whether the sample means were significantly different from each other and the sample data meets the assumptions of One‐way ANOVA analysis based on the Normal distribution test and Homogeneity of variance test. Additionally, Permutational Multivariate Analysis of Variance (PERMANOVA) was employed to evaluate the significance of dissimilarities among groups. The statistical analyses and visual representations were generated utilizing the Biozeron Cloud Platform (http://www.cloud.biomicroclass.com/CloudPlatform).

## 3 RESULTS

### 3.1 Fish species composition based on eDNA

The comprehensive raw species abundance data acquired through eDNA metabarcoding analysis can be located in Table A2. This table provides a breakdown of the abundance data at the species level for each sampling site, which forms the foundation for subsequent analyses in the manuscript. A total of 132 Molecular Operational Taxonomic Units (MOTUs) were identified across the four seasons, encompassing 8 orders, 24 families, and 85 genera. Within this dataset, Table 1 highlights the presence of 8 species classified as state‐level protected fish, 27 species endemic to the upper Yangtze River, and 7 species designated as Chongqing municipal key protected fish.

**TABLE 1:** List of fish species detected in the Reserve based on environmental DNA metabarcoding.

Order	Family	Genus	Species	The detection situation of fish in different seasons at each site					
				MFT	QJK	DJT	GZT	SJ	SPH
Cypriniformes	Cyprinidae	*Acheilognathus*	*A. barbatus*	A	A	A	A	A	A
			*A. gracilis*		A		A		
			*A. omeiensis*★	D				A	
			*A. chankaensis*	C	C	C	C	C	C
			*A. macropterus*	C	C	C	C	C	C
		*Megalobrama*	*M. pellegrini*★	ACD	ACD	ACD	ACD	ACD	ACD
			*M. amblycephala*▲	AC	AC	AC	AC	AC	AC
		*Erythroculter*	*E. dabryi*	ACD	ACD	ACD	ACD	ACD	ACD
			*E. ilishaeformis*	ABD	ABD	ABD	ABD	ABD	ABD
		*Culter*	*C. alburnus*	AC	AC	AC	AC	AC	AC
			*C. erythropterus*		C				
		*Hemiculter*	*H. leucisculus*	ACD	ACD	ABCD	ACD	ACD	ACD
			*H. tchangi*★	ABCD	ABCD	ABCD	ABCD	ABCD	ABCD
		*Hemiculterella*	*H. sauvagei*★					A	A
		*Pseudohemiculter*	*P. dispar*	ABCD	ABD	ABCD	ABD	ABCD	ABD
		*Pseudolaubuca*	*P. sinensis*	AC	AC	AC	AC	AC	AC
			*P. engraulis*	ABC	ABC	ABC	ABC	ABC	ABCD
		*Coreius*	*C. guichenoti*●★	A					
			*C. heterodon*	A	A	A	A	A	A
		*Pseudorasbora*	*P. parva*▲	ABD	ABD	ABD	ABD	ABD	ABD
		*Rhinogobio*	*R. typus*	ABCD	ABCD	ABCD	ABCD	ABCD	ABCD
			*R. ventralis*●★	ABCD	ABCD	ABCD	ABCD	ABCD	ABCD
		*Saurogobio*	*S. dabryi*	AC	AC	AC	AC	AC	AC
			*S. dumerili*	BD	BD	BD	D	D	BD
		*Squalidus*	*S. argentatus*	ACD	ACD	ACD	ACD	ACD	ACD
			*S. wolterstorffi*	A	A	A	A	A	A
		*Spinibarbus*	*S. sinensis*	ABCD	ABCD	ABCD	ABCD	ABCD	ABCD
		*Ctenopharyngodon*	*C. idellus*	ABCD	ABCD	ABCD	ABCD	ABCD	ABCD
		*Squaliobarbus*	*S. curriculus*	ABCD	ABCD	ABD	ABD	ABCD	ABCD
		*Carassius*	*C. auratus*	ABCD	ABCD	ABCD	ABCD	ABCD	ABCD
			*C. cuvieri*	C	C	C	C	C	C
			*C. gibelio*	C	C	C	C	C	C
		*Hypophthalmichthys*	*H. molitrix*	ACD	ACD	ACD	ACD	ACD	ACD
			*H. nobilis*	ABCD	ABCD	ABCD	ABCD	ABCD	ABCD
		*Mylopharyngodon*	*M. piceus*	A	A	A	A	AC	AC
		*Hemibarbus*	*H. labeo*	C	AC	AC	AC	AC	AC
			*H. maculatus*	AB	AB	AB	AB	AB	AB
		*Abbottina*	*A. rivularis*	ABC	BC	BC	BC	AC	BC
		*Gobiobotia*	*G. filifer*	A	A	A	A	A	A
		*Xenophysogobio*	*X. nudicorpa*★	A	A	A	A		
		*Procypris*	*P. rabaudi*●★	AC	AC	AC	AC	AC	AC
		*Cyprinus*	*C. carpio*	CD	CD	CD	CD	CD	CD
		*Sinilabeo*	*S. tungting*	A		A		A	A
			*S. rendahli*★	C	C	C	C	C	C
		*Sarcocheilichthys*	*S. sinensis*	A	A	A			
			*S. nigripinnis*	A	A	A	A	A	A
		*Platysmacheilus*	*P. nudiventris*★					A	A
		*Sinibrama*	*S. taeniatus*★	A	A	A	A	A	A
		*Parabramis*	*P. pekinensis*	A	AC	AC	A	AC	A
		*Ancherythroculter*	*A. nigrocauda*★	ABD	AD	AD	AD	AD	AD
			*A. wangi*★	ABCD	ABCD	ABCD	ABCD	ABCD	ABCD
		*Pseudobrama*	*P. simoni*	AB	ABC	AB	AB	AB	AB
		*Xenocypris*	*X. argentea*	A	A	A	A	A	
			*X. yunnanensis*★	D	D	D	D	D	D
		*Distoechodon*	*D. tumirostris*	AC	AC	AC	AC	AC	AC
		*Acrossocheilus*	*A. monticolus*★	AB	AB	AB	AB	AB	AB
		*Rhodeus*	*R. sinensis*	ABD	AB	ABD	ABD	ABD	AB
			*R. ocellatus*	AC	AC	AC	AC	AC	AC
		*Microphysogobio*	*M. kiatingensis*	ABD	ABCD	BD	ABD	ABD	ABD
		*Danio*	*D. rerio*	ACD	ACD	ACD	ACD	ACD	ACD
		*Discogobio*	*D. yunnanensis*	B	B	AB	A	A	
		*Elopichthys*	*E. bambusa*	AC	C	C	C	AC	C
		Ochetobius	*O. elongatus*■	A	A	A	A	A	A
		*Schizothorax*	*S. wangchiachii*★	AB	AB	AB		A	AB
		*Anabarilius*	*A. brevianalis*★	C	C	C	C	C	C
			*A. liui*★	C	C	C	C	C	C
		*Onychostoma*	*O. macrolepis*●		C	C			
		*Opsariichthys*	*O. bidens*	C	C	C	C	C	C
		*Zacco*	*Z. platypus*	C	C	C	C	C	C
	Catostomidae	*Myxocyprinus*	*M. asiaticus*●	C	AC	C	AC	C	C
	Cobitidae	*Leptobotia*	*L. microphthalma*★■	ACD	ACD	ACD	AC	AC	ACD
			*L. rubrilaris*★	A	A	A	A	A	
			*L. taeniaps*■	ABD	ABD	ABD	ABD	ABD	ABD
		*Sinibotia*	*S. superciliaris*■	ACD	ACD	ACD	ACD	ACD	ACD
			*S. reevesae*★	A	A	A	A	A	A
		*Misgurnus*	*M. mizolepis*	BD	BD	BD	BD	BD	BD
		*Paramisgurnus*	*P. dabryanus*	ABCD	ABCD	ABCD	ABCD	ABCD	ABCD
		*Paracobitis*	*P. potanini*★	ABC	ABC	ABC	ABC	ABC	ABC
			*P. variegatus*	A					
		*Parabotia*	*P. fasciata*	ABD	AD	AD	ABD	ABD	ABD
		*Triplophysa*	*T. anterodorsalis*	ABD	ABD	ABD	ABD	ABD	ABD
			*T. rosa*■	ABD	ABD	ABD	ABD	ABD	ABD
			*T. stenura*	AB	AB	B	AB	AB	AB
		*Schistura*	*S. fasciolata*					A	A
	Homalopteridae	*Lepturichthys*	*L. fimbriata*	A	A	A	A	A	A
		*Sinogastromyzon*	*S. sichangensis*★	ABCD	ACD	ABCD	ACD	ABCD	ABCD
		*Jinshaia*	*J. abbreviata*★	C	C	C	C	C	C
Acipenseriformes	Acipenseridae	*Acipenser*	*A. sinensis*●	AD	AD	AD	AD	AD	AD
			*A. dabryanus*●★	C	C	C	C	C	C
			*A. schrenckii*▲	B	B	B	B	B	B
Perciformes	Channidae	*Channa*	*C. argus*	AD	AD	AD	ABD	ABD	ABD
			*C. asiatica*	D	D	D	D	D	D
	Gobiidae	*Rhinogobius*	*R. cliffordpopei*	ABCD	ABCD	ABCD	ABCD	ABCD	ABCD
			*R. brunneus*	AB	AB	AB	AB	BA	AB
			*R. giurinus*	C	C	C	C	C	C
		*Mugilogobius*	*M. myxodermus*				A		
	Serranidae	*Siniperca*	*S. chuatsi*	ABCD	ABCD	ABCD	ABCD	ABCD	ABCD
			*S. knerii*	ABCD	ACD	ACD	ABCD	ACD	ABCD
			*S. scherzeri*	C	C		C	C	C
	Centrarchidae	*Micropterus*	*M. salmoides*▲	CD	ABCD	ACD	CD	BCD	ACD
	Eleotridae	*Micropercops*	*M. swinhonis*	ABD	ABD	ABD	ABD	ABD	ABD
	Cichlidae	*Oreochromis*	*O*. sp.—▲	AD	AD	AD	AD	AD	AD
			*O. niloticus*▲	C	AC	AC	C	ABC	AC
	*Mastacembelidae*	*Mastacembelus*	*M. armatus*	C	C				
		*Sinobdella*	*S. sinensis*	B	B	B	B	AB	A
Siluriformes	Sisoridae	*Glyptothorax*	*G. sinensis*	ABCD	ABCD	ABCD	ABCD	ABCD	ABCD
	Bagridae	*Hemibagrus*	*H. macropterus*	ACD	ACD	ACD	ACD	ACD	ACD
		*Leiocassis*	*L. longirostris*	AD	D	AD	AD	AD	AD
		*Pseudobagrus*	*P. brevicaudatus*	ABCD	ABCD	ABCD	ABCD	ABCD	ABCD
			*P. pratti*■	AC	AC	AC	AC	AC	AC
			*P. albomarginatus*	D	D	D	D	D	D
			*P. crassilabris*	ACD	ACD	ACD	ACD	ACD	ACD
		*Pelteobagrus*	*P. fulvidraco*	ABCD	ABCD	ABCD	ABCD	ABCD	ABCD
			*P. nitidus*	ACD	ACD	ACD	ACD	ACD	ACD
			*P. vachellii*	ABD	ABD	ABD	ABD	ABD	ABD
			*P. eupogon*	D	D	D	D	D	D
	Siluridae	*Silurus*	*S. asotus*	ABCD	ABCD	ABCD	ABCD	ABCD	ABCD
			*S. meridionalis*	ABCD	ABCD	ABCD	ABCD	ABCD	ABCD
	Amblycipitidae	*Liobagrus*	*L. marginatus*■	ABCD	ABCD	ABCD	ABCD	ABCD	ABCD
			*L. marginatoides*★	AC			AC	AC	C
			*L. kingi*●★						A
	Ictaluridae	*Ictalurus*	*I. punctatus*▲		A			A	A
		*Ameiurus*	*A. nebulosus*						A
	Clariidae	*Clarias*	*C*. sp.*—*	A	A	A	A	A	A
Salmoniformes	Salangidae	*Neosalanx*	*N. tangkahkeii*	AB	AB	AB	AB	AB	AB
			*N. taihuensis*▲	D	D	D	D	D	D
		*Protosalanx*	*P. hyalocranius*▲		B	A	AB		A
	Salmonidae	*Oncorhynchus*	*O. mykiss*▲	AB	AB	AB	AB	AB	AB
Synbranchiformes	Synbranchidae	*Monopterus*	*M. albus*						A
Cyprinodontiformes	Poeciliidae	*Gambusia*	*G. affinis*▲	A	A	A	A	A	A
	Adrianichthyidae	*Oryzias*	*O. latipes*	C	C	C	C	C	C
Beloniformes	Hemiramphidae	*Hyporhamphus*	*H. intermedius*	A					
Total 8	24	85	132	120	119	115	115	118	116

*Note*: “—” means the highest resolution not reach to this taxa level; ●, National protected fishes; ★, Endemic fish in the upper reaches of the Yangtze River; ■, Key protected fishes in Chongqing; ▲, Alien fishes; A, fish composition in summer; B, fish composition in autumn; C, fish composition in winter; D, fish composition in spring.

At the order level (Table 2), the highest species richness was observed in Cypriniformes, accounting for 65.91% of the total number of species, followed by Siluriformes (14.40%), Perciformes (11.36%), and other orders exhibited lower species richness. Cyprinidae was the most diverse family, comprising 69 species (52.27% of the total), followed by Cobitidae (10.61%), Bagridae (7.58%), and other families displayed lower species diversity. At the species level, *Carassius auratus*, *Cyprinus carpio*, *Hemiculter tchangi*, and *Ctenopharyngodon idellus* demonstrated a notable relative sequence abundance consistently throughout the year, collectively representing over 50% of the total abundance. Additionally, 24 species, such as *Hemiculter leucisculus*, *Pseudolaubuca engraulis*, and *H. tchangi*, were detected in all four seasons, while the remaining 108 species were exclusively detected during specific seasons.

**TABLE 2:** Species composition of fish at the family and order level based on traditional methods and environmental DNA.

Order	Family	eDNA			Traditional		
		Species (*N*)	Percentage (*P*)	Total	Species (*N*)	Percentage (*P*)	Total
Cypriniformes	Cyprinidae	69	52.27%	N = 87 P = 65.91%	56	54.37%	N = 73 P = 70.87%
	Catostomidae	1	0.76%		1	0.97%	
	Cobitidae	14	10.61%		12	11.65%	
	Homalopteridae	3	2.27%		4	3.83	
Acipenseriformes	Acipenseridae	3	2.27%	N = 3 P = 2.27%	1	0.97%	N = 1 P = 0.97%
Perciformes	Channidae	2	1.52%	N = 15 P = 11.36%	1	0.97%	N = 9 P = 8.74%
	Gobiidae	4	3.03%		1	0.97%	
	Serranidae	3	2.27%		4	3.83%	
	Centrarchidae	1	0.76%		1	0.97%	
	Eleotridae	1	0.76%		1	0.97%	
	Cichlidae	2	1.52%		1	0.97%	
	Mastacembelidae	2	1.52%		0	0%	
Siluriformes	Sisoridae	1	0.76%	N = 19 P = 14.40%	2	1.94%	N = 16 P = 15.53%
	Bagridae	10	7.58		8	7.77%	
	Siluridae	2	1.52%		2	1.94%	
	Amblycipitidae	3	2.27%		3	2.91%	
	Ictaluridae	2	1.52%		1	0.97%	
	Clariidae	1	0.76%		0	0%	
Salmoniformes	Salangidae	1	0.76%	N = 4 P = 3.03%	2	1.94%	N = 2 P = 1.94%
	Salmonidae	3	2.27%		0	0%	
Synbranchiformes	Synbranchidae	1	0.76%	N = 1 P = 0.76%	1	0.97%	N = 1 P = 0.97%
Cyprinodontiformes	Poeciliidae	1	0.76%	N = 2 P = 1.52%	1	0.97%	N = 1 P = 0.97%
	Adrianichthyidae	1	0.76%		0	0%	
Beloniformes	Hemiramphidae	1	0.76%	N = 1 P = 0.76%	0	0%	0
Total		132	100%		103	100%	

**FIGURE 2:**
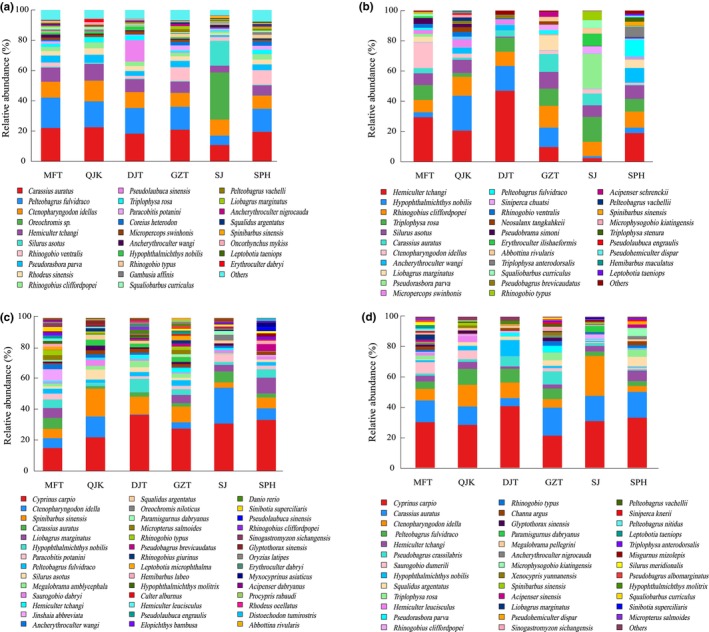
Species composition of fish at the species level based on sequence abundance (a: fish composition in summer; b: fish composition in autumn; c: fish composition in winter; d: fish composition in spring).

Further analysis of the relative sequence abundance indicated variations in fish community composition at the species level across the four seasons (Figure 2). Specifically, *C. auratus* and *Pelteobagrus fulvidraco* exhibited the greatest relative sequence abundance during the summer, as confirmed by Kruskal‐Wallis test, significantly higher than in the autumn and winter seasons (*p* < .05). Moreover, these species were evenly distributed across all sampling sites. In autumn, *H. tchangi* exhibited the highest relative sequence abundance, particularly at the DJT site, as determined by Kruskal‐Wallis test, relative sequence abundance was significantly higher in summer and autumn than in winter *p* < .05). Conversely, *C. carpio* displayed relativelv high sequence abundance in winter and spring, surpassing the autumn and summer seasons significantly (*p* < .01). as determined through Kruskal‐Wallis test.

The permanova analysis indicated no significant variation in fish community composition among sites (*R* ^2^ = .133, *p* = .99). However, there are differences in the composition and abundance of prominent species across different sampling sites (Table A3). In the MFT, QJK, and GZT sites, *C. auratus* exhibited the highest sequence abundance, accounting for 13.4%, 40.22%, and 14.82% respectively, followed by *C. carpio* with percentages of 13.04%, 10.3%, and 12.5% respectively. In the DJT and SPH sites, *C. carpio* exhibited the highest sequence abundance, accounting for 18.59% and 17.41% respectively, followed by *H. tchangi* (14.77%) in DJT and *C. auratus* (13.13%) in SPH. In the SJ site, *C. idellus* displayed the highest sequence abundance (18.72%), followed by *C. carpio* (18.25%), *C. auratus* (11.31%), and *Oreochromis* sp. (10.44%). Notably, at the QJK sampling site, the proportion of *C. auratus* (40.22%) sequences is significantly higher compared to the other sites.

### 3.2 Distribution of alien and endemic fish

**FIGURE 3:**
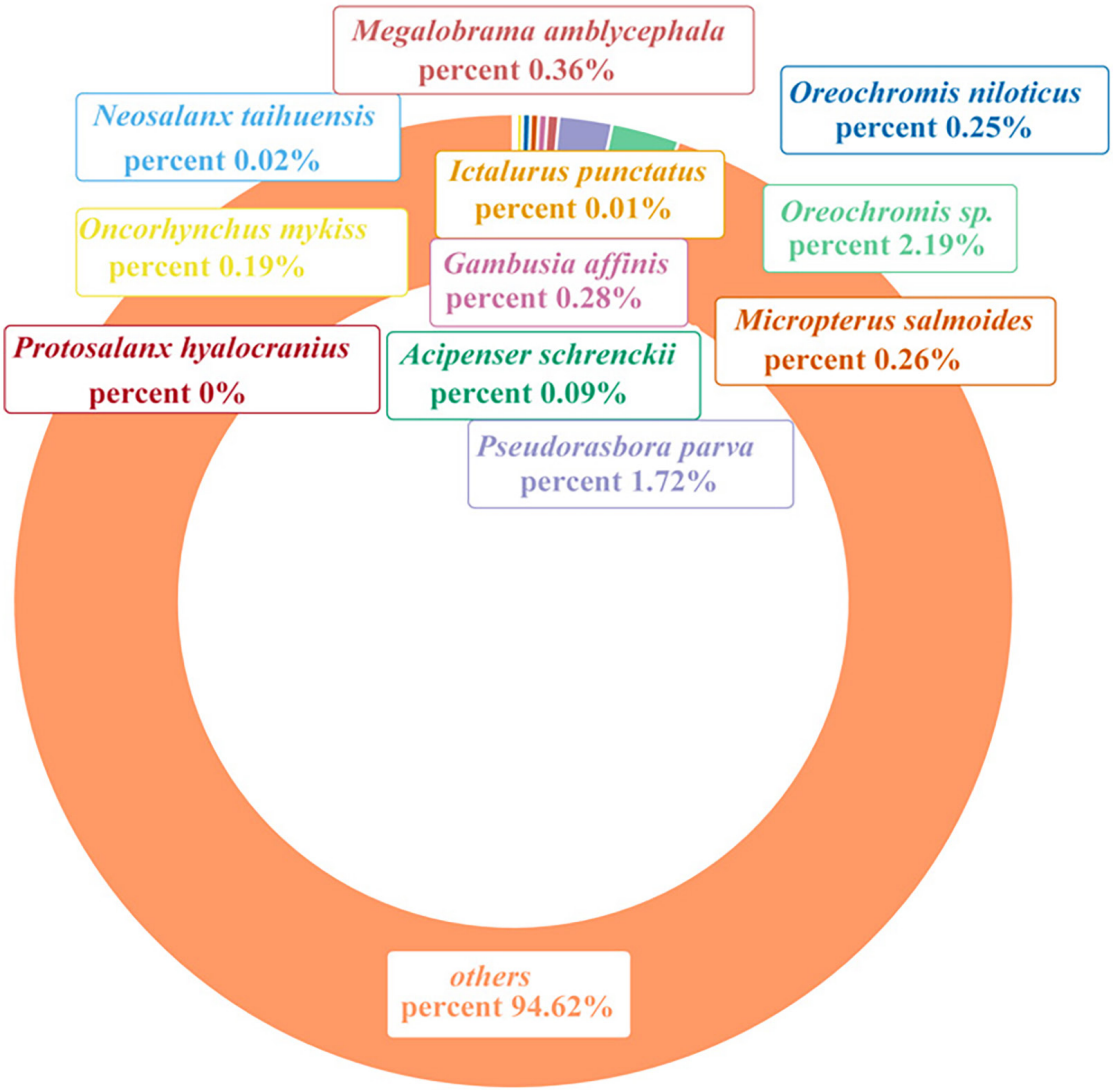
The proportion of alien fish species in the overall fish community structure based on sequence abundance.

A total of 11 alien species, such as *Pseudorasbora parva*, *Oreochromis niloticus*, and *Acipenser schrenckii*, were detected with eDNA. According to the analysis of the proportion of eDNA sequence abundance of alien fish in the overall of fish (Figure 3), *Oreochromis* sp. constituted the highest proportion, reaching 2.19% followed by *P. parva* at 1.72%. Conversely, *Protosalanx hyalocranius* exhibited the lowest proportion, amounting to a mere 0.001%.

**FIGURE 4:**
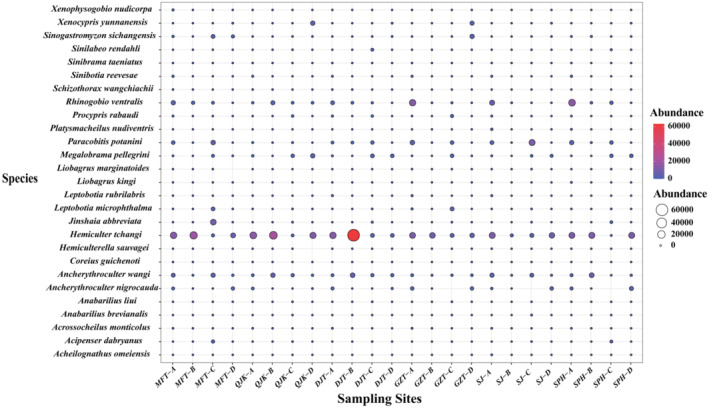
The distribution of endemic fish based on sequence abundance (a: in summer; b: in autumn; c: in winter; d: in spring).

A total of 27 endemic fish species, such as *Megalobrama pellegrini*, *H. tchangi*, *Xenophysogobio nudicorpa*, were detected based on eDNA. According to the abundance distribution of endemic fish species (Figure 4), *H. tchangi* exhibited the highest relative sequence abundance throughout the basin. Notably, the DJT site recorded the maximum number of sequences in October, surpassing those of other endemic fish species significantly. Conversely, *Coreius guichenoti* displayed the lowest relative sequence abundance, with a minimal number of sequences detected in the MFT, SJ and SPH sites in June.

### 3.3 Species coverage rate based on eDNA

Over the past decade, approximately 103 species of aquatic fauna have been collected from the Chongqing section of the Reserve using traditional methods such as gillnets and cast nets (Gao et al., 2013; He et al., 2018; Tian et al., 2016). Compared with eDNA, the proportion of species number showed the same trend at the family level (Table 2). And Cyprinidae had the most species, followed by Cobitidae and Bagridae. In addition, Mastacembelidae, Clariidae, Salmonidae, Adrianichthyidae and Hemiramphidae were detected only by eDNA. At the species level, the comparison of eDNA metabarcoding and traditional methods revealed some differences. Traditional methods evidenced that *Coreius heterodon*, *C. guichenoti* and *Rhinogobio ventralis* dominate species (Table A1), whereas the eDNA method showed a relatively higher sequence abundance of *C. carpio*, *Carassius cuvieri*, and *H. tchangi*.

**FIGURE 5:**
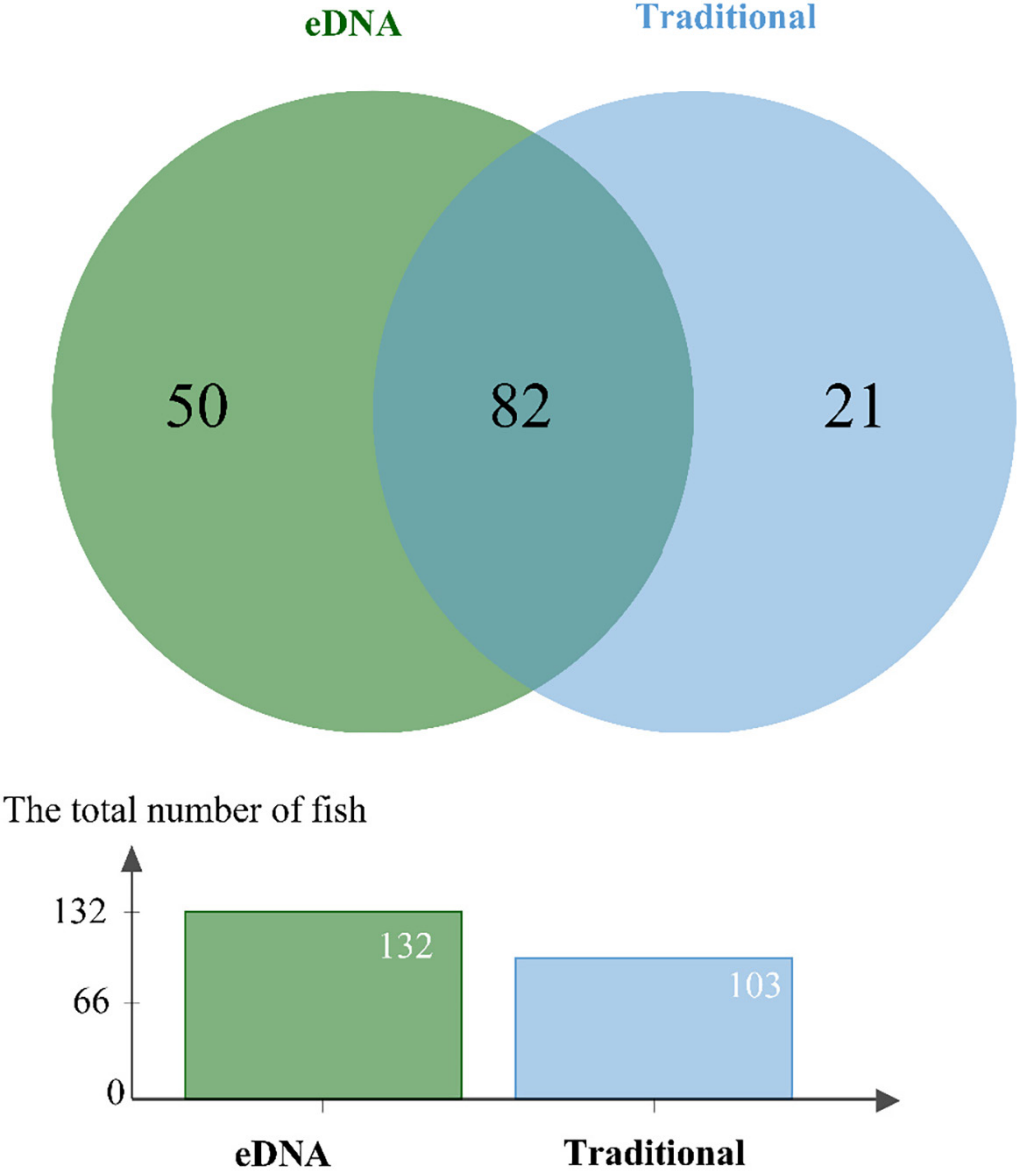
Venn diagram of species detection comparison based on eDNA and traditional methods.

Based on 103 fish species collected by traditional methods, we calculated the annual coverage rate. A total of 132 fish species were found in this study, and 82 fish species were consistent with the results of traditional methods in recent years (Figure 5). This indicated that the overall coverage rate was high, which was 79.61%. In addition, 50 species of fish were detected only by eDNA, such as *Megalobrama amblycephala*, *Sinilabeo tungting* and *Leptobotia microphthalma*. Conversely, 26 fish species were solely detected by traditional methods only, such as *Culter mongolicus*, *Hemiculter bleekeri* and *Rhinogobio cylindricus*.

### 3.4 Analysis of fish diversity in the reserve

The diversity indices are displayed in Table 3, and their mean values were analyzed using a one‐way ANOVA. The richness index was found to be significantly higher in summer (*p* < .05), indicating a greater community richness during this season. Conversely, autumn exhibited the lowest community richness (*p* < .05). The patterns observed in the fluctuations of Shannon and Simpson diversity indices, which reflect community diversity, remained consistent across various sampling sites. Notably, the diversity of fish communities in summer was significantly greater than that in spring (*p* < .05). Additionally, the Pielou index did not show significant seasonal variations. When comparing the diversity indices among different sampling sites, the mean richness index was highest at the QJK site, indicating the highest community richness at this location. The consistent patterns in the changes of Shannon and Simpson indices were also observed across different sites, with the fish community diversity at GZT significantly higher than at SJ and DJT sites (*p* < .05). Furthermore, the Pielou index indicated that species distribution was most evenly spread at the GZT site, while the SJ site exhibited the least even distribution. In summary, fish community diversity and species richness were highest during the summer, and the SJ site displayed the lowest diversity among the sampled sites.

**TABLE 3:** The diversity index of variety sites based on environmental DNA.

Sample	Richness					Shannon					Simpson					Pielou				
	Summer	Autumn	Winter	Spring	Mean	Summer	Autumn	Winter	Spring	Mean	Summer	Autumn	Winter	Spring	Mean	Summer	Autumn	Winter	Spring	Mean
MFT	98	50	79	66	73.25^a^	3.92	3.53	4.09	3.78	3.83^ab^	0.12	0.12	0.06	0.15	0.11^ab^	0.59	0.63	0.71	0.62	0.64^ab^
QJK	98	58	86	67	77.25^a^	3.75	3.97	3.92	3.44	3.77^ab^	0.12	0.09	0.12	0.16	0.12^ab^	0.57	0.68	0.61	0.57	0.61^abc^
DJT	95	57	81	61	73.5^a^	3.93	2.99	3.72	3.32	3.49^a^	0.11	0.2	0.17	0.17	0.16^a^	0.6	0.51	0.59	0.56	0.57^ac^
GZT	95	54	82	67	74.5^a^	4.12	3.59	4.34	4.02	4.02^b^	0.1	0.10	0.10	0.10	0.1^b^	0.63	0.62	0.68	0.66	0.65^b^
SJ	100	53	76	68	74.25^a^	3.67	3.43	3.43	3.12	3.41^a^	0.15	0.12	0.19	0.20	0.17^a^	0.55	0.6	0.55	0.51	0.55^c^
SPH	102	56	75	64	74.25^a^	4.19	4	3.71	3.23	3.78^ab^	0.09	0.08	0.14	0.20	0.13^ab^	0.63	0.69	0.6	0.55	0.62^abc^
Mean	98^a^	54.67^b^	79.83^c^	65.5^d^		3.93^a^	3.59^ab^	3.87^ab^	3.49^b^		0.115^a^	0.118^ab^	0.13^ab^	0.16^b^		0.6^a^	0.622^a^	0.623^a^	0.58^a^	

*Note*: For different means (distinguishing between rows and columns) within the same diversity index, the presence of the same lowercase letter indicates nonsignificant differences (*p* > .05), while the absence of identical letters signifies significant differences (*p* < .05).

**FIGURE 6:**
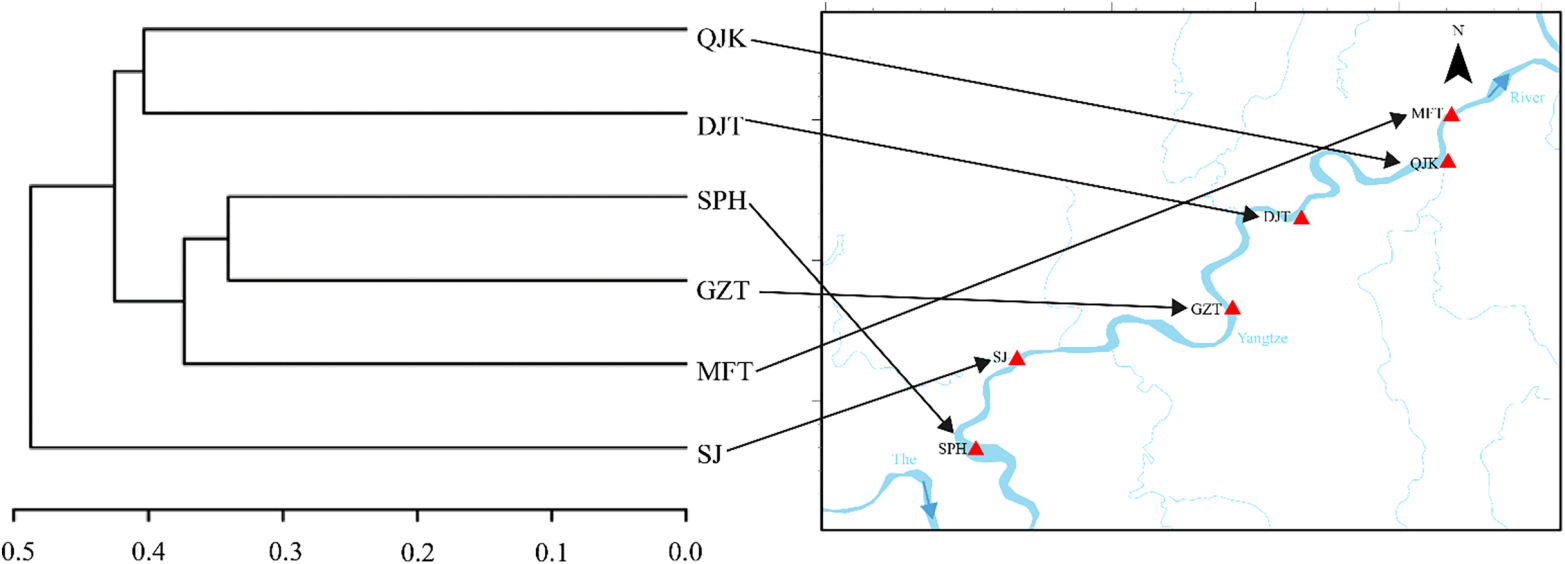
UPGMA cluster analysis diagram of 6 sampling sites based on eDNA detection.

**FIGURE 7:**
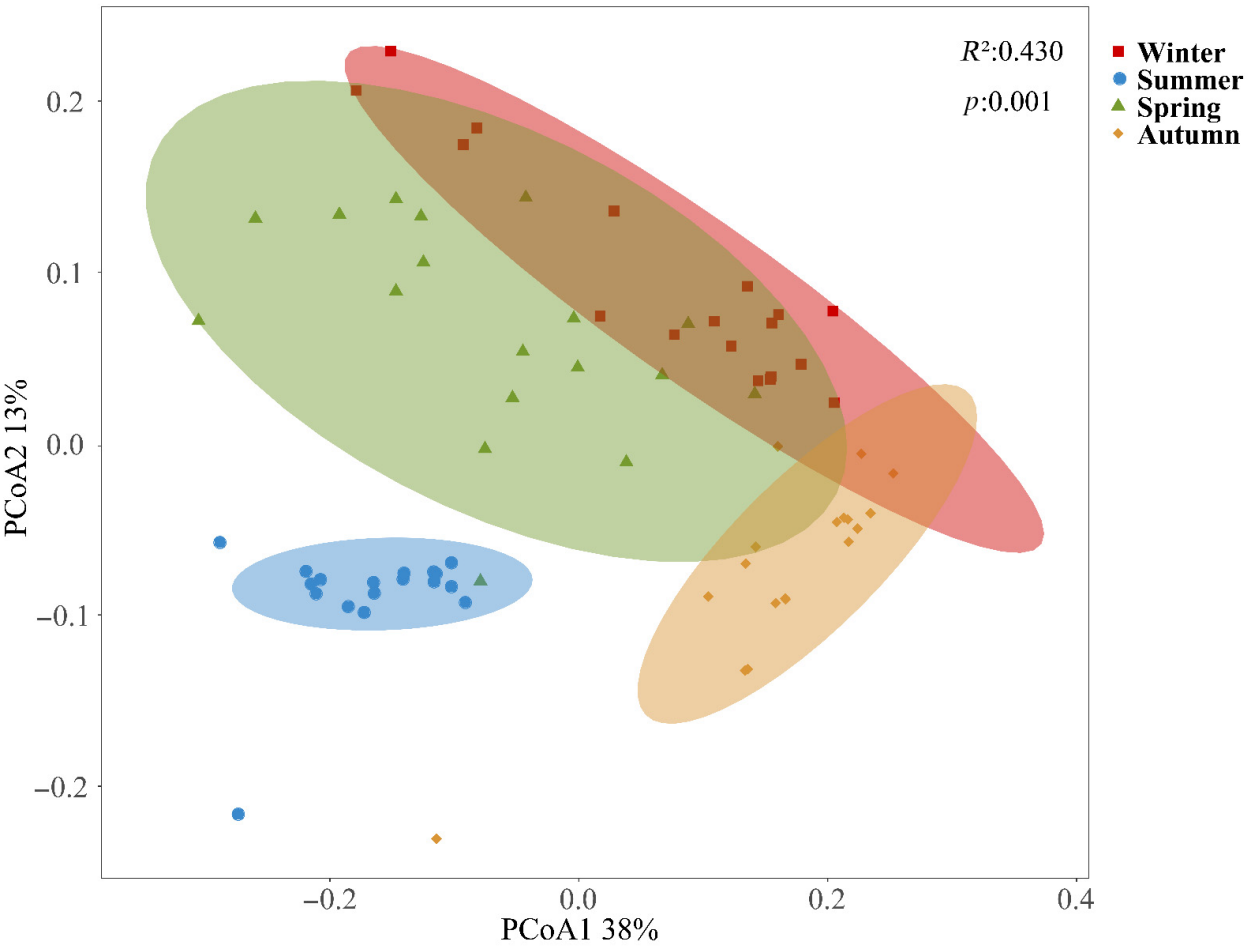
Analysis of fish principal coordinates based on Bray‐Curtis distance matrix.

The UPGMA cluster diagram shows that SPH, GZT, and MFT had the most similar species composition, followed by QJK and DJT and then SJ (Figure 6). Furthermore, the PCoA analysis (Figure 7), incorporating the results of the PERMANOVA analysis (*R* ^2^ = .430, *p* = .001), based on sequence abundance, demonstrated a statistically significant distinction in fish community structure across seasons. This finding highlights the substantial influence of seasonality on the fish community (*p* < .01). Additionally, pairwise comparisons were conducted among fish community compositions in different seasons, based on the PERMANOVA analysis, revealing significant differences among them (*p* < .01). These findings suggest the existence of significant temporal and spatial variations in the fish community structure in the Chongqing section of the Reserve.

## 4 DISCUSSION

### 4.1 Fish diversity in the reserve

The eDNA analysis yielded a total of 132 fish species, offering a more comprehensive understanding of fish diversity in comparison to our initial eDNA investigation (Wang et al., 2022). The utilization of repeated seasonal sampling and meticulous spatial sampling techniques in this study significantly contributed to the augmented comprehensiveness of the monitoring outcomes. In contrast to conventional methodologies, alterations in the dominant fish populations in the Reserve were observed. This disparity can be attributed to the protracted duration of traditional surveys (7–10 years), during which fish stocks were persistently influenced by human activities (Gao et al., 2013; He et al., 2018; Tian et al., 2016). Notably, there has been a notable decline in the populations of economically significant species, such as *C. heterodon*. Moreover, the observed disparities may be influenced by variations in detection methods, including sampling season and frequency (Shaw et al., 2016; Zhang et al., 2020).

The detection of certain alien fishes can be attributed to factors such as the proximity of fish farms situated at the Reserve's boundary, where inadequate measures to prevent species escape may be in place. Additional factors that may contribute to the introduction of invasive species include artificial discharge, fishing, and transportation activities (Ba & Chen, 2012). Other potential contributors include artificial discharge, as well as fishing and transportation activities (Ba & Chen, 2012). It is important to note that the detection of these invasive species is primarily based on DNA spillover rather than the actual spillover of individual organisms. This implies that although the eDNA of these species is present in the adjacent waters, their actual presence and establishment beyond the aquaculture ponds may be constrained. However, it does not rule out the possibility of the presence of individual organisms of these non‐native fish species in the water body. Further investigation is warranted to accurately assess the extent of their spread and potential ecological impacts. In this study, *Oreochromis* sp. comprises the most significant proportion, accounting for up to 2.19%. *Oreochromis* sp. is an omnivorous fish with high adaptability. It exhibits rapid growth and reproductive rates, along with robust territorial and juvenile care behavior. Therefore, *Oreochromis* sp. may encroach on the habitats of native species and disrupt the original structure and function of ecosystems, which cause the decline and even extinction of indigenous fish populations. In order to mitigate the influence of alien fish on the fish diversity in the Reserve, it is advisable for the relevant authorities to implement necessary measures (Liu et al., 2021). For instance, constructing facilities around fish farms adjacent to the Reserve to hinder isolation and escape of fish species could be considered. Additionally, it is recommended to formulate appropriate policies that discourage citizens from releasing fish into the Yangtze River without proper authorization.

The survey detected 27 species of endemic fish in the upper Yangtze River, with *H. tchangi* displaying the highest relative sequence abundance across the entire basin, consistent with previous resource monitoring in the Yangtze River basin. Historically, economically valuable fish species like *C. guichenoti*, *C. heterodon*, and *P. fulvidraco* held dominance in this section. However, this survey revealed that the proportion of *C. guichenoti* within the fish community amounted to less than 0.1%. This phenomenon is posited to be linked to factors such as the development of terraced hydropower and overfishing. *C. guichenoti* is a benthic species with a preference for inhabiting swiftly flowing rivers and streams. However, with the development of the upstream Jinsha River main tributaries such as the gradient hydropower, the hydrological conditions have changed and *C. guichenoti* population has been decreasing. Conversely, prior to the implementation of the great protection of the Yangtze River, *C. guichenoti*, serving as a vital economic fish in the upper Yangtze River, was frequently subject to selective artificial fishing practices, leading to population decreases. Based on the urgency and seriousness of the conservation of *C. guichenoti*, there is a strong call for relevant authorities to take action. For example, the establishment of a dedicated conservation facility for *C. guichenoti* within the Yangtze River and the implementation of scientifically standardized stocking and releasing practices should be considered.

### 4.2 Temporal and spatial distribution of fish in the reserve

This study substantiates the utility of freshwater eDNA analysis in tracking the fluctuating composition of fish species in the Yangtze River throughout different seasons (Figure 5, Table 3). Meanwhile, the study confirmed the recent findings of Stoeckle et al. (2017) that eDNA samples reflect seasonal variations in fish composition.

The results of the PCoA analysis revealed significant variations in the fish population structure within the Chongqing section of the Reserve across different seasons (*p* < .01), indicating strong temporal dynamics (Littlefair et al., 2021; Mächler et al., 2021). The substantial dissimilarity observed in the fish community structure across the four seasons can be attributed to various ecological factors. Firstly, fluctuations in temperature throughout different seasons lead to alterations in water temperature, directly impacting the distribution, behavior, and reproductive activities of fish species (McAdam et al., 2005). Secondly, seasonal variations in food availability and resource availability can induce shifts in fish species composition and abundance (Littlefair et al., 2021; Mächler et al., 2021). Additionally, the breeding and migration patterns of specific fish species are often closely tied to particular seasons, further contributing to the observed distinctions (Tao et al., 2008). Furthermore, environmental parameters such as water flow, dissolved oxygen levels, and water chemistry also experience seasonal changes, influencing the habitat preferences and survival of diverse fish species. (Flood et al., 2021; Yacobi et al., 1993). It is important to note that these factors interact with each other and with other environmental variables in complex ways, influencing the dynamics of the fish community across seasons. Thus, it is recommended that future research consider these factors for a comprehensive understanding. Understanding these relationships and the underlying mechanisms driving the observed variations in fish composition is crucial for the effective management and conservation of fish populations within the protected area.

The diversity analysis showed that species richness and species diversity were the highest across the Reserve in summer. Simultaneously, the highest number of endemic fish species were also detected in summer, including *Leptobotia rubrilaris*, *C. heterodon*, and *C. guichenoti*. These results suggest that seasonal variations of fish species in the Reserve may be related to factors like fish community behavior, seasonal temperature difference, and hydrological regime (Wang et al., 2022). The months from May to July encompasses the breeding season of most fish species in the upper Yangtze River (Liu, 2018), including those like *C. auratus* and *P. fulvidraco*, which exhibited higher sequence abundance during the summer in this study. Hence, it is conceivable that the enhanced detection during summer sampling might be attributed to a greater availability of eDNA sources for certain species (e.g., gametes and fish fry) (Stewart, 2019). In addition, the water flow velocity in the upper Yangtze River is faster during summer tin han other seasons. The 25 fish species detected only in summer mostly inhabit environments with faster flow velocity, such as *Lepturichthys fimbriata*, *Ochetobius elongatus*, and *S. tungtin*. It is plausible that these fish species, which are adapted to fast currents, might have migrated to more upstream areas beyond the Reserve Chongqing section during other seasons (Yang et al., 2012). Relevant studies have indicated that due to the elevated water temperatures, the feeding intensity, activity, and reproductive levels of fish species increase, leading to higher fish species diversity during the spring and summer seasons compared to the autumn and winter seasons (Zhou et al., 2016). This bears a certain resemblance to our research findings, as the Reserve in the Chongqing section experiences an average seasonal temperature difference of 13.4°C (Zhang et al., 2014), with the highest temperatures occurring in summer, which is also reflected in the eDNA data's diversity analysis, revealing a more diverse fish community during this season. Therefore, the fish community in the Reserve Chongqing section is mainly influenced by seasonal migrations and changes in population size, altering the temporal and spatial distribution characteristics of the fish community.

The diversity of fish not only showed seasonal differences but also showed differences among basins (Table 3). The fluctuations in eDNA relative sequence abundance and fish species detection at various sampling sites may indicate alterations in habitat utilization and distribution (Stoeckle et al., 2017). For instance, Stoeckle et al. (2017) demonstrated that discrepancies in fish eDNA detections corresponded to habitat preferences identified through conventional surveys. Despite some similarities in species composition among different sites in the same seasons (Figure 2), there were variations in the annual changes of fish diversity indexes across different sampling sites (Table 3). Notably, the watershed of the SJ site in the Reserve exhibited the lowest fish diversity. Furthermore, the UPGMA cluster diagram revealed a significant dissimilarity in the community structure of SJ compared to other sampling sites (Figure 6). This observed pattern could potentially be attributed to the interplay of natural environmental factors and anthropological activities in the Chongqing section of the Reserve (Wang et al., 2022). Moreover, variations in the total nitrogen concentration within river systems possess the capacity to exert an impact on the spatial distribution of fish, as evidenced by prior investigations (Shi et al., 2018). The SJ site, situated in close proximity to the Songji Ancient Town, is susceptible to potential contamination originating from urban sewage. Additionally, the shoreline of this site is characterized by extensive pebble beaches featuring limited vegetation, minimal nutrient influx from terrestrial sources, shallow water depths, and rapid water flow. Consequently, these factors may contribute to reduced fish activity within this area, thereby resulting in diminished eDNA detection of fish species. Moreover, the QJK sampling site, situated at the confluence of the Qijiang River and the Yangtze River, exhibits a substantial water surface area and a sluggish flow rate, accompanied by numerous coastal depressions that contribute to a notable level of habitat heterogeneity. Nutrient runoff from the surrounding surface runoff enters the Qijiang River and subsequently flows into the mainstream of the Yangtze River. This creates a favorable environment for various fish species, particularly for species such as *C. auratus* (relative sequence abundance of 40.22%) and *C. carpio* (relative sequence abundance of 10.3%). As a result, the QJK site demonstrates the highest community richness, aligning with previous research findings (Wang et al., 2022). The intricate and varied environment generated by the merging of the Qijiang River and the Yangtze River is likely accountable for the observed high species richness at this location (Stoeckle et al., 2017).

### 4.3 Suggestions on fish diversity conservation

In this study, the utilization of eDNA metabarcoding enabled the detection of a total of 132 fish species, among which were *C. guichenoti*, *Ancherythroculter wangi*, *Paracobitis potanini*, and various other endemic fish species inhabiting the upper Yangtze River. However, it is noteworthy that certain rare and endemic fish species, namely *Percocypris pingi*, *Leptobotia elongata*, and *Onychostoma angustistomata*, which possess historical records, were not detected. This absence may be attributed to their limited presence in the Yangtze River ecosystem. Additionally, the investigation revealed the presence of 11 alien fish species, including *Gambusia affinis*, *Ictalurus punctatus*, and *O. niloticus*, which pose a potential threat to the survival of indigenous fish populations. If appropriate actions are not implemented, alterations in fish diversity and community composition will have an impact on the overall well‐being of the aquatic ecosystem (Ma, 2020). It is highly recommended that the relevant governing bodies prioritize two crucial measures to protect fish resources in the Yangtze River Basin: The requirement for rigorous oversight and logical resolution of ecological security issues in the reserve is highlighted, including the prudent construction of artificial fish nests and reefs, as well as the vigilant monitoring of biological invasion in the basin (Wang, 2021). Additionally, it is imperative to enhance scientific research efforts and establish adaptive management mechanisms, such as conducting comprehensive assessments on the abundance, population dynamics, and level of threat posed by fish species in the Yangtze River Basin, undertaking fundamental research on fish biology and ecology, and intensifying the surveillance of aquatic ecosystems' health (Cao, 2017).

### 4.4 Limitations of eDNA metabarcoding

While eDNA offers several advantages compared to traditional methods for fish species detection, it is not without limitations. Methodological factors such as sampling techniques, choice of primers, and reference databases can significantly influence the results obtained from eDNA analysis (Shu, 2022). To achieve optimal results, an eDNA metabarcoding approach should strike a balance between being conservative and moderately different (Chen et al., 2016). The “Tele02” primer, an improved version derived from the “MiFish‐U” primer, demonstrates enhanced taxa resolution (Taberlet et al., 2018). However, certain studies have indicated that even with this primer, it remains challenging to differentiate closely related species based on the amplified fragments (e.g., *Misgurnus mizolepis* and *Misgurnus anguillicaudatus*) (Shu, 2022; Xiong et al., 2014; Yao et al., 2020). In order to address the limitations of the current eDNA primers, which have a short amplification fragment and result in low species resolution (Hänfling et al., 2016; Valentini et al., 2016; Yamamoto et al., 2017; Zhang et al., 2020), it is imperative to employ a multi‐primers combined eDNA technology in future studies.

In addition, the coverage rate of eDNA is also influenced by the choice of a comparison database that includes representative OTU sequences. This comparison database can either be a publicly available database such as MitoFish (http://mitofish.aori.u‐tokyo.ac.jp/) or a self‐established database based on historical data. Insufficient or imprecise gene sequences of species in either self‐built or public databases can potentially result in false negative outcomes. Moreover, the limited length of amplicons can readily lead to species‐matching failures and false positive results. Consequently, the reliability of species identification is directly contingent upon the integrity and quality of the reference database (Li et al., 2019). In this study, the abundance of unidentified sequences may be attributed to the inadequate sequence length and the absence of molecular data for certain fish species in the self‐built database, particularly those from the Yangtze River.

The fish species detected in this study did not cover all the fish species present in the basin. Nevertheless, the utilization of environmental DNA (eDNA) yielded a higher number of fish species detections compared to the outcomes obtained through the traditional method, resulting in a commendable overall coverage rate. It is important to acknowledge that each method possesses its own set of limitations and advantages. In the case of eDNA, it has the ability to detect DNA released by fish dispersed throughout the study area, whereas the traditional method necessitates the capture of individual fish, including those that are rare and protected. Consequently, the eDNA method exhibits greater sensitivity and poses minimal harm. However, it is imperative to integrate eDNA with conventional methodologies in order to thoroughly evaluate variables such as the abundance of fish stocks, the distribution of sizes, and the composition of age groups within the designated research area. Furthermore, disparities in results can arise due to variations in sampling time and frequency. In summary, this substantiates the efficacy of eDNA in detecting the diversity of fish species in the upper reaches of the Yangtze River. Notably, the utilization of eDNA metabarcoding exclusively unveiled the presence of *Acipenser dabryanus*, *Procypris rabaudi*, and an additional 55 fish species. This observation suggests that eDNA metabarcoding not only facilitates prompt and efficient fish monitoring but also exhibits a heightened level of sensitivity.

## 5 CONCLUSION

eDNA metabarcoding demonstrates significant potential for biomonitoring freshwater biodiversity. In this study, the application of eDNA metabarcoding in the Chongqing section of the Reserve yields a comprehensive inventory of fish species, detecting a total of 132 species across various replicates, sampling sites, and seasons. A comparative analysis between the diversity assessment derived from our study and that of a recent fish catch highlights the efficacy of eDNA metabarcoding in facilitating expedited and effective fish monitoring, while also exhibiting a remarkable level of sensitivity. Moreover, the study provides evidence that eDNA metabarcoding can serve as a valuable tool for monitoring temporal fluctuations in fish composition within freshwater ecosystems. However, it is important to acknowledge that the eDNA method currently lacks standardized protocols. In summary, given the implementation of a decade‐long fishing prohibition in the Yangtze River basin, the utilization of eDNA metabarcoding as an innovative and transformative approach will greatly contribute to the enhancement of fish diversity management and conservation efforts. Additionally, this novel method offers a means to evaluate the impact of invasive alien species on fishery resources and aquatic ecosystems.

## AUTHOR CONTRIBUTIONS

**Yanjun Shen:** Funding acquisition (equal); methodology (equal); writing – review and editing (equal). **Yufeng Zhang:** Formal analysis (equal); software (equal); supervision (equal); writing – original draft (equal); writing – review and editing (equal). **Ruli Cheng:** Formal analysis (equal). **Wei Wang:** Investigation (equal). **Cong Duan:** Investigation (equal). **Zhihao Liu:** Investigation (equal). **Qiliang Chen:** Investigation (equal). **Yingwen Li:** Funding acquisition (equal); investigation (equal); project administration (equal). **Meng Wang:** Data curation (equal); investigation (equal); resources (equal); software (equal); supervision (equal); writing – original draft (equal). **Yang Luo:** Data curation (equal).

## CONFLICT OF INTEREST STATEMENT

The authors declare that they have no conflict of interest.

## ETHICS STATEMENT

The experiments involving animals in this study were conducted in accordance with the Laboratory Animal Management Principles of China. All experimental protocols were approved by the Ethics Committee of Chongqing Normal University.

## Data Availability

The reference sequences and the raw data are available on NCBI (https://dataview.ncbi.nlm.nih.gov) under the following accession numbers SAMN31723196‐SAMN31723219 and PRJNA837764, respectively.

## APPENDIX A

**TABLE A1:** Types of catches fish in Chongqing section of Reserve based on traditional methods.

Order	Family	Genus	Species	National protected fishes	Endemic fish in the upper reaches of the Yangtze River	Key protected fishes in Chongqing	Alien fish species
Cypriniformes	Cyprinidae	*Acheilognathus*	*A. chankaensis*				
			*A. gracilis*				
			*A. macropterus*				
			*A. omeiensis*				
		*Megalobrama*	*M. pellegrini*		★		
		*Chanodichthys*	*C. dabryi*				
		*Culter*	*C. alburnus*				
			*C. mongolicus*				
		*Cultrichthys*	*C. erythropterus*				
		*Hemiculter*	*H. leucisculus*				
			*H. tchangi*		★		
			*H. bleekeri*				
		*Hemiculterella*	*H. sauvagei*		★		
		*Pseudolaubuca*	*P. sinensis*				
			*P. aengraulis*				
		*Coreius*	*C. guichenoti*	●	★		
			*C. heterodon*				
		*Pseudorasbora*	*P. parva*				▲
		*Rhinogobio*	*R. typus*				
			*R. cylindricus*		★		
			*R. ventralis*		★		
		*Saurogobio*	*S. dabryi*				
		*Squalidus*	*S. argentatus*				
		*Spinibarbus*	*S. sinensis*				
		*Ctenopharyngodon*	*C. idellus*				
		*Squaliobarbus*	*S. curriculus*				
		*Carassius*	*C. auratus*				
		*Hypophthalmichthys*	*H. molitrix*				
		*Cyprinus*	*C. carpio*				
		*Mylopharyngodon*	*M. piceus*				

		*Hemibarbus*	*H. labeo*				
			*H. maculatus*				
		*Abbottina*	*A. rivularis*				
		*Gobiobotia*	*G. filifer*				
		*Xenophysogobio*	*X. boulengeri*		★		
			*X. nudicorpa*		★		
		*Onychostoma*	*O. sima*				
		*Cirrhinus*	*C. mrigala*				▲
		*Procypris*	*P. rabaudi*		★	■	
		*Sinilabeo*	*S. rendahli*				
		*Sarcocheilichthys*	*S. sinensis*				
			*S. nigripinnis*				
		*Platysmacheilus*	*P. nudiventris*		★		
		*Opsariichthys*	*O. bidens*				
		*Sinibrama*	*S. wuitypus*				
			*S. taeniatus*		★		
		*Parabramis*	*P. pekinensis*				
		*Ancherythroculter*	*A. nigrocauda*		★		
			*A. wangi*		★		
			*A. kurematsui*		★		
		*Pseudobrama*	*P. simoni*				
		*Xenocypris*	*X. davidi*				
			*X. argentea*				
		*Distoechodon*	*D. tumirostris*				
*Rhodeus*	*R. sinensis*				
Catostomidae	*Myxocyprinus*	*M. asiaticus*	●			

	Cobitidae	*Leptobotia*	*L. elongata*	●	★	■	
			*L. rubrilaris*		★	■	
			*L. taeniaps*				
		*Sinibotia*	*S. superciliaris*				
		*Paramisgurnus*	*P. dabryanus*				
		*Misgurnus*	*M. anguillicaudatus*				
		*Paracobitis*	*P. potanini*		★		
			*P. variegatus*				
		*Parabotia*	*P. fasciata*				
			*P. bimaculata*		★		
		*Triplophysa*	*T. bleekeri*				
			*T. brevicauda*				
	Homalopteridae	*Lepturichthy*	*L. fimbriata*				
		*Jinshaia*	*J. abbreviata*		★		
			*J. sinensis*		★	■	
*Metahomaloptera*		*M. omeiensis*			■	
Acipenseriformes	Acipenseridae	*Acipenser*	*A. dabryanus*	●	★		
Perciformes	Channidae	*Channa*	*C. argus*				
	Gobiidae	*Rhinogobius*	*R. giurinus*				
	Serranidae	*Siniperca*	*S. chuatsi*				
			*S. knerii*				
			*S. scherzeri*				
		*Lateolabrax*	*L. japonicus*				
	Centrarchidae	*Micropterus*	*M. salmoides*				▲
	Eleotridae	*Hypseleotris*	*H. swinhonis*				
	Cichlidae	*Oreochromis*	*O. mossambicus*				▲
Siluriformes	Sisoridae	*Glyptothorax*	*G. sinensis*				
			*G. fukiensis*				
	Bagridae	*Hemibagrus*	*H. macropterus*				
		*Leiocassis*	*P. crassilabris*				
			*L. longirostris*				
		*Pseudobagrus*	*P. emarginatus*				
			*P. pratti*				
		*Pelteobagrus*	*P. fulvidraco*				
			*P. nitidus*				
			*P. vachellii*				
			*P. eupogon*				
	Siluridae	*Silurus*	*S. asotus*				
			*S. meridionalis*				
	Amblycipitidae	*Liobagrus*	*L. marginatus*				
			*L. marginatoides*		★		
			*L. nigricauda*				▲
	Ictaluridae	*Ictalurus*	*I. punctatus*				▲
Salmoniformes	Salangidae	*Neosalanx*	*N. taihuensis*				
		*Protosalanx*	*P. haylocranius*				▲
Synbranchiformes	Synbranchidae	*Monopterus*	*M. albus*				
Cyprinodontiformes	Poeciliidae	*Gambusia*	*G. affinis*				▲
Total 7 19 69			103	4	22	5	8

*Note*: ●, National protected fishes; ★, Endemic fish in the upper reaches of the Yangtze River; ■, Key protected fishes in Chongqing; ▲, Alien fishes.

**TABLE A2:** Annual fish composition and sequence number of each sampling site of the Reserve in Chongqing section based on environmental DNA.

Species	Read counts of each species at each site																							
	MFT				QJK				DJT				GZT				SJ				SPH			
	Summer	Autumn	Winter	Spring	Summer	Autumn	Winter	Spring	Summer	Autumn	Winter	Spring	Summer	Autumn	Winter	Spring	Summer	Autumn	Winter	Spring	Summer	Autumn	Winter	Spring
*Abbottina rivularis*	1	18	3	0	0	25	7	0	0	17	4	0	0	2580	991	0	82	1859	5	0	0	29	12	0
*Acanthorhodeus gracilis*	0	0	0	0	19	0	0	0	0	0	0	0	1	0	0	0	0	0	0	0	0	0	0	0
*Acheilognathus barbatus*	3	0	0	0	220	0	0	0	3	0	0	0	95	0	0	0	2	0	0	0	1	0	0	0
*Acheilognathus omeiensis*	0	0	0	1	0	0	0	0	0	0	0	0	0	0	0	0	45	0	0	0	0	0	0	0
*Acanthorhodeus chankaensis*	0	0	1	0	0	0	2	0	0	0	3	0	0	0	2	0	0	0	670	0	0	0	6	0
*Acheilognathus macropterus*	0	0	2	0	0	0	3	0	0	0	2	0	0	0	12	0	0	0	909	0	0	0	5	0
*Acipenser sinensis*	675	0	0	2236	626	0	0	13	12	0	0	14	743	0	0	341	346	0	0	544	254	0	0	3497
*Acipenser schrenckii*	0	12	0	0	0	15	0	0	0	12	0	0	0	2962	0	0	0	14	0	0	0	16	0	0
*Acipenser dabryanus*	0	0	1485	0	0	0	14	0	0	0	11	0	0	0	7	0	0	0	14	0	0	0	909	0
*Anabarilius brevianalis*	0	0	10	0	0	0	9	0	0	0	7	0	0	0	9	0	0	0	99	0	0	0	11	0
*Anabarilius liui*	0	0	3	0	0	0	15	0	0	0	18	0	0	0	18	0	0	0	5	0	0	0	3	0
*Acrossocheilus monticolus*	36	7	0	0	39	6	0	0	57	2	0	0	87	15	0	0	38	3	0	0	29	5	0	0
*Ameiurus nebulosus*	0	0	0	0	0	0	0	0	0	0	0	0	0	0	0	0	0	0	0	0	1	0	0	0
*Ancherythroculter nigrocauda*	1048	1	0	1707	833	0	0	39	1237	0	0	229	1931	0	0	1829	392	0	0	2046	1265	0	0	3325
*Ancherythroculter wangi*	2810	95	3782	393	768	4310	1623	366	955	5077	1436	1413	923	138	371	568	3768	123	2817	160	1185	5713	30	284
*Sinilabeo rendahli*	0	0	4	0	0	0	14	0	0	0	911	0	0	0	6	0	0	0	5	0	0	0	144	0
*Sinilabeo tungting*	2	0	0	0	0	0	0	0	2	0	0	0	0	0	0	0	1	0	0	0	3	0	0	0
*Carassius auratus*	31,197	2208	8492	21,582	31,252	1465	1073	306,323	28,009	5176	2969	8493	29,143	10,891	2204	31,797	26,707	4007	15,728	37,478	30,465	260	2229	26,677
*Carassius cuvieri*	0	0	3	0	0	0	17	0	0	0	20	0	0	0	14	0	0	0	20	0	0	0	1	0
*Carassius gibelio*	0	0	14	0	0	0	13	0	0	0	867	0	0	0	18	0	0	0	38	0	0	0	8	0
*Channa argus*	460	0	0	3242	405	0	0	1032	562	0	0	2929	10	98	0	27	116	1	0	604	366	1	0	4147
*Channa asiatica*	0	0	0	161	0	0	0	2	0	0	0	604	0	0	0	1	0	0	0	2	0	0	0	2
*Clarias* sp.	294	0	0	0	244	0	0	0	157	0	0	0	135	0	0	0	5	0	0	0	311	0	0	0
*Coreius guichenoti*	3	0	0	0	0	0	0	0	0	0	0	0	0	0	0	0	0	0	0	0	0	0	0	0
*Coreius heterodon*	1433	0	0	0	49	0	0	0	610	0	0	0	3271	0	0	0	1744	0	0	0	4745	0	0	0
*Ctenopharyngodon idellus*	14,851	11,052	7559	11,449	18,856	2827	10,113	36,300	16,148	179	364	16,442	12,723	2136	4008	9631	26,326	1530	51,545	59,515	13,497	707	6384	6128
*Cyprinus carpio*	0	0	16,549	45,163	0	0	15,470	71,622	0	0	37,425	64,814	0	0	26,005	36,464	0	0	65,965	69,470	0	0	27,426	51,642
*Culter alburnus*	8	0	16	0	13	0	15	0	11	0	441	0	20	0	873	0	40	0	2496	0	14	0	612	0
*Culter erythropterus*	0	0	0	0	0	0	17	0	0	0	0	0	0	0	0	0	0	0	0	0	0	0	0	0
*Danio rerio*	6	0	7	403	1	0	11	3	2	0	2272	2	61	0	1105	4	27	0	6	416	1	0	11	392
*Discogobio yunnanensis*	0	1	0	0	0	1	0	0	1	1	0	0	1	0	0	0	1	0	0	0	0	0	0	0
*Distoechodon tumirostris*	1	0	5	0	1	0	7	0	1	0	6	0	99	0	6	0	267	0	4	0	1	0	1346	0
*Elopichthys bambusa*	1	0	2398	0	0	0	18	0	0	0	9	0	0	0	8	0	1	0	8	0	0	0	1542	0
*Erythroculter dabryi*	120	0	5	6	3036	0	7	1153	33	0	2436	3	37	0	29	2	184	0	6	3	3	0	5	2
*Erythroculter ilishaeformis*	18	771	0	4	114	30	0	5	90	31	0	986	113	39	0	2	6	4170	0	3	99	27	0	2
*Gambusia affinis*	1948	0	0	0	2169	0	0	0	529	0	0	0	779	0	0	0	1782	0	0	0	2655	0	0	0
*Glyptothorax sinensis*	303	405	4	2087	13	3	2113	4884	689	2	14	46	563	3	120	4077	98	3	279	26	580	2	3	20
*Gobiobotia filifer*	25	0	0	0	1	0	0	0	91	0	0	0	2	0	0	0	89	0	0	0	35	0	0	0
*Hemibagrus macropterus*	1	0	4	9	81	0	3	1733	43	0	3	1	3	0	4	5	382	0	1345	6	228	0	7	2
*Hemibarbus labeo*	0	0	2218	0	1	0	24	0	5	0	86	0	81	0	20	0	7	0	804	0	51	0	1861	0
*Hemibarbus maculatus*	31	7	0	0	210	1158	0	0	659	11	0	0	565	15	0	0	1392	9	0	0	790	5	0	0
*Hemiculter leucisculus*	36	0	1961	2366	19	0	686	10,112	19	3	421	119	20	0	44	246	102	0	1277	509	14	0	9	850
*Hemiculter tchangi*	13,228	19,029	1263	5715	15,226	21,799	827	11,809	13,069	62,918	2768	2507	10,254	8940	2950	4564	11,099	1246	3156	8390	10,480	11,040	69	10,846
*Hemiculterella sauvagei*	0	0	0	0	0	0	0	0	0	0	0	0	0	0	0	0	2	0	0	0	1	0	0	0
*Hypophthalmichthys molitrix*	220	0	2186	18	361	0	18	101	498	0	1788	3079	181	0	262	12	27	0	719	16	86	0	30	9
*Hypophthalmichthys nobilis*	2073	2094	6635	1947	1488	24,483	1449	7673	3988	21,941	9237	17,292	1455	11,832	3632	2515	570	643	4080	1721	712	2094	4676	974
*Hyporhamphus intermedius*	12	0	0	0	0	0	0	0	0	0	0	0	0	0	0	0	0	0	0	0	0	0	0	0
*Jinshaia abbreviata*	0	0	9120	0	0	0	41	0	0	0	154	0	0	0	26	0	0	0	22	0	0	0	762	0
*Ictalurus punctatus*	0	0	0	0	1	0	0	0	0	0	0	0	0	0	0	0	5	0	0	0	272	0	0	0
*Leiocassis longirostris*	10	0	0	57	0	0	0	57	4	0	0	236	5	0	0	150	1	0	0	33	10	0	0	4
*Leptobotia microphthalma*	10	0	2532	13	3	0	21	1	3	0	23	1	178	0	2841	0	4	0	15	0	11	0	19	1
*Leptobotia rubrilabris*	4	0	0	0	2	0	0	0	173	0	0	0	198	0	0	0	143	0	0	0	0	0	0	0
*Leptobotia taeniops*	777	4	0	3280	21	4	0	13	249	3	0	9	570	3	0	10	484	6	0	413	1621	697	0	6
*Lepturichthys fimbriata*	20	0	0	0	3	0	0	0	5	0	0	0	415	0	0	0	52	0	0	0	480	0	0	0
*Liobagrus kingi*	0	0	0	0	0	0	0	0	0	0	0	0	0	0	0	0	0	0	0	0	71	0	0	0
*Liobagrus marginatoides*	1	0	6	0	0	0	0	0	0	0	0	0	2	0	4	0	1	0	8	0	0	0	9	0
*Liobagrus marginatus*	681	2250	7683	4969	40	83	148	19	480	125	110	21	2770	8981	5233	1026	1226	128	9547	16	2011	3066	8777	10
*Mastacembelus armatus*	0	0	1	0	0	0	92	0	0	0	0	0	0	0	0	0	0	0	0	0	0	0	0	0
*Megalobrama amblycephala*	64	0	2060	0	332	0	1836	0	7	0	4456	0	5	0	2659	0	1	0	1532	0	1	0	54	0
*Megalobrama pellegrini*	70	0	995	48	443	0	1912	4042	114	0	2663	2164	22	0	2022	28	94	0	521	1018	17	0	1783	2010
*Micropercops swinhonis*	2062	1696	0	4	2219	5839	0	3	2879	4182	0	6	1914	486	0	2	677	104	0	3	1461	911	0	1
*Microphysogobio kiatingensis*	2	13	0	33	7	1764	9	38	0	13	0	29	1	16	0	31	128	25	0	108	14	6	0	8422
*Micropterus salmoides*	0	0	3793	1546	5	2	604	2	54	0	522	3	0	0	2755	2	0	2	27	3	43	0	37	1
*Misgurnus mizolepis*	0	4	0	14	0	5	0	13	0	559	0	297	0	12	0	15	0	6	0	3210	0	436	0	7
*Monopterus albus*	0	0	0	0	0	0	0	0	0	0	0	0	0	0	0	0	0	0	0	0	2	0	0	0
*Mugilogobius myxodermus*	0	0	0	0	0	0	0	0	0	0	0	0	81	0	0	0	0	0	0	0	0	0	0	0
*Mylopharyngodon piceus*	63	0	0	0	3	0	0	0	34	0	0	0	3	0	0	0	70	0	8	0	7	0	4	0
*Myxocyprinus asiaticus*	0	0	9	0	1	0	10	0	0	0	8	0	34	0	6	0	0	0	4	0	0	0	2451	0
*Neosalanx taihuensis*	0	0	0	830	0	0	0	3	0	0	0	2	0	0	0	2	0	0	0	2	0	0	0	4
*Neosalanx tangkahkeii*	141	35	0	0	5	3419	0	0	362	430	0	0	335	1956	0	0	210	55	0	0	633	29	0	0
*Ochetobius elongatus*	13	0	0	0	2	0	0	0	4	0	0	0	2	0	0	0	13	0	0	0	3	0	0	0
*Oncorhynchus mykiss*	1281	2	0	0	1682	2	0	0	1045	327	0	0	880	87	0	0	796	4	0	0	504	3	0	0
*Oreochromis niloticus*	0	0	24	0	2	0	25	0	1	0	26	0	0	0	53	0	544	7	8329	0	2	0	36	0
*Oreochromis* sp.	15	0	0	1	216	0	0	2	13	0	0	1	178	0	0	4	76,994	0	0	493	319	0	0	1
*Onychostoma macrolepis*	0	0	0	0	0	0	2	0	0	0	1	0	0	0	0	0	0	0	0	0	0	0	0	0
*Opsariichthys bidens*	0	0	43	0	0	0	2898	0	0	0	2540	0	0	0	3094	0	0	0	21	0	0	0	22	0
*Oryzias latipes*	0	0	1159	0	0	0	57	0	0	0	16	0	0	0	1244	0	0	0	12	0	0	0	16	0
*Pseudobagrus albomarginatus*	0	0	0	1708	0	0	0	69	0	0	0	67	0	0	0	1509	0	0	0	13	0	0	0	14
*Parabotia fasciata*	1022	2	0	10	224	0	0	1185	635	0	0	4	429	1	0	5	422	2	0	1	629	171	0	2
*Parabramis pekinensis*	4	0	0	0	1	0	2	0	1	0	7	0	5	0	0	0	18	0	1	0	2	0	0	0
*Paracobitis potanini*	2201	7	4550	0	102	7	104	0	804	810	1782	0	4722	11	2207	0	2649	3	11,225	0	3967	96	2023	0
*Paracobitis variegatus*	21	0	0	0	0	0	0	0	0	0	0	0	0	0	0	0	0	0	0	0	0	0	0	0
*Paramisgurnus dabryanus*	218	2	1123	37	256	3	242	31	127	324	25	121	356	4	1392	650	17	4	5294	9289	115	117	36	19
*Pelteobagrus fulvidraco*	28,419	875	3802	7382	23,666	1161	1609	26,533	25,914	72	2892	14,429	21,199	2169	3597	12,106	15,497	72	47	6654	23,736	6589	1970	5054
*Pelteobagrus nitidus*	96	0	3	2512	202	0	298	1116	182	0	7	240	212	0	4	33	50	0	1	22	116	0	6	13
*Pelteobagrus vachelli*	298	17	0	228	436	2541	0	2229	730	22	0	62	2753	16	0	2861	2994	30	0	13	1906	14	0	11
*Pelteobagrus eupogon*	0	0	0	3	0	0	0	27	0	0	0	1	0	0	0	3	0	0	0	1	0	0	0	703
*Platysmacheilus nudiventris*	0	0	0	0	0	0	0	0	0	0	0	0	0	0	0	0	361	0	0	0	81	0	0	0
*Procypris rabaudi*	118	0	10	0	4	0	448	0	220	0	391	0	20	0	1291	0	104	0	6	0	36	0	5	0
*Protosalanx hyalocranius*	0	0	0	0	0	2	0	0	1	0	0	0	1	1	0	0	0	0	0	0	1	0	0	0
*Pseudobagrus brevicaudatus*	677	23	92	5	26	1669	32	2	21	2227	1019	677	715	50	1890	2	355	31	45	1	890	23	3946	1
*Leiocassis crassilabris*	37	0	14	2188	166	0	17	4320	77	0	14	10,415	32	0	1171	14,899	27	0	1305	4419	142	0	1229	1278
*Pseudobagrus pratti*	393	0	11	0	138	0	12	0	536	0	10	0	474	0	1849	0	359	0	1762	0	230	0	10	0
*Pseudobrama simoni*	512	2493	0	0	400	2335	14	0	208	41	0	0	120	40	0	0	53	48	0	0	483	229	0	0
*Pseudohemiculter dispar*	242	6	80	520	135	273	0	29	29	287	19	1109	17	8	0	801	257	12	33	24	27	693	0	3222
*Pseudolaubuca engraulis*	545	7	2241	0	9	1331	23	0	635	11	20	0	258	12	1907	0	288	16	15	0	880	8	25	8
*Pseudolaubuca sinensis*	182	0	12	0	227	0	11	0	21,707	0	10	0	468	0	24	0	229	0	1123	0	388	0	2179	0
*Pseudorasbora parva*	6600	1221	0	126	7433	72	0	70	4940	60	0	3922	5819	572	0	7548	2982	11,686	0	1184	5930	308	0	872
*Rhinogobio typus*	854	273	3920	1966	184	153	923	71	995	46	36	61	3005	21	2140	5200	1772	3041	22	2768	3288	194	93	2725
*Rhinogobio ventralis*	3984	1608	873	4	328	3294	683	673	2666	746	292	1	12,830	40	185	3	6325	53	9	3	15,025	441	1847	3
*Rhinogobius brunneus*	213	8	0	0	1145	142	0	0	78	520	0	0	23	26	0	0	63	8	0	0	276	7	0	0
*Rhinogobius cliffordpopei*	3718	5301	129	1119	5296	13,207	105	3524	4533	12,631	2001	1809	3150	13,240	16	29	2989	4907	891	6407	5399	6175	11	18
*Rhinogobius giurinus*	0	0	17	0	0	0	1514	0	0	0	648	0	0	0	2114	0	0	0	2230	0	0	0	30	0
*Rhodeus ocellatus*	4	0	4	0	65	0	14	0	125	0	1281	0	4	0	18	0	2	0	643	0	2	0	4	0
*Rhodeus sinensis*	4206	2	0	1	5866	1	0	0	5009	3	0	1	4279	424	0	1	2983	3	0	1	5028	180	0	0
*Sarcocheilichthys nigripinnis*	2	0	0	0	125	0	0	0	2	0	0	0	1	0	0	0	57	0	0	0	1	0	0	0
*Sarcocheilichthys sinensis*	53	0	0	0	6	0	0	0	1	0	0	0	0	0	0	0	0	0	0	0	0	0	0	0
*Saurogobio dumerili*	466	1	0	11,259	66	40	0	14,911	968	2	0	104	297	0	0	3675	436	0	0	1720	1284	32	0	1958
*Saurogobio dabryi*	0	0	811	0	0	0	3065	0	0	0	1122	0	0	0	682	0	0	0	4317	0	0	0	1707	0
*Schistura fasciolata*	0	0	0	0	0	0	0	0	0	0	0	0	0	0	0	0	5	0	0	0	5	0	0	0
*Schizothorax wangchiachii*	13	1	0	0	8	7	0	0	11	9	0	0	0	0	0	0	1	0	0	0	1	1	0	0
*Silurus asotus*	745	5065	1833	7	671	9315	4841	684	631	1049	3328	299	481	10,246	1567	1	40,696	3979	31	160	578	5225	1333	3
*Silurus meriordinalis*	383	1	1149	3110	297	1	8	8	198	1	6	8	486	41	4	7	281	1	4	297	185	1	11	2
*Sinibotia reevesae*	295	0	0	0	162	0	0	0	79	0	0	0	198	0	0	0	145	0	0	0	266	0	0	0
*Sinibotia superciliaris*	172	0	3322	9	363	0	17	8	190	0	24	4	378	0	12	6	166	0	6	2502	264	0	6	6
*Sinibrama taeniatus*	3	0	0	0	4	0	0	0	6	0	0	0	5	0	0	0	2	0	0	0	5	0	0	0
*Siniperca chuatsi*	400	32	1162	19	583	364	2317	12	568	403	1079	6	788	3393	1418	10	404	2432	3207	5	382	371	30	7
*Siniperca knerii*	1	1	6	1482	2	0	49	19	3	0	18	34	5	3	39	2021	2	0	916	13	1	1	1	1749
*Siniperca scherzeri*	0	0	3	0	0	0	2	0	0	0	0	0	0	0	2	0	0	0	7	0	0	0	1	0
*Sinobdella sinensis*	0	4	0	0	0	11	0	0	0	1234	0	0	0	6	0	0	2	1	0	0	1	0	0	0
*Sinogastromyzon sichangensis*	331	1	2780	1377	7	0	13	22	3	1	14	47	7	0	8	4112	1	1	8	21	68	151	6	13
*Spinibarbus sinensis*	1081	469	7266	739	993	10	13,439	4238	304	8	12,109	27	286	12	10,104	24	3057	14	7768	2004	521	1434	6003	19
*Squalidus argentatus*	1379	0	24	811	355	0	2129	5858	657	0	1777	3546	1566	0	381	6322	986	0	4173	1070	1554	0	22	10,001
*Squalidus wolterstorffi*	6	0	0	0	3	0	0	0	5	0	0	0	3	0	0	0	3	0	0	0	5	0	0	0
*Squaliobarbus curriculus*	2272	1111	4	16	2303	29	1	805	1307	26	0	19	887	29	0	2026	1327	2691	21	11	1609	163	1	8
*Triplophysa anterodorsalis*	186	20	0	11	408	27	0	9	412	14	0	17	631	24	0	2114	552	35	0	13	1072	3955	0	1543
*Triplophysa rosa*	2988	6345	0	3812	4811	2656	0	63	4677	12,390	0	132	1662	10,392	0	8675	1738	8492	0	106	4401	4875	0	7442
*Triplophysa stenura*	1	7	0	0	2	6	0	0	0	6	0	0	1	11	0	0	1	12	0	0	1	1654	0	0
*Xenocypris argentea*	14	0	0	0	91	0	0	0	1	0	0	0	79	0	0	0	1	0	0	0	0	0	0	0
*Xenophysogobio nudicorpa*	201	0	0	0	1	0	0	0	3	0	0	0	2	0	0	0	0	0	0	0	0	0	0	0
*Xenocypris yunnanensis*	0	0	0	50	0	0	0	4127	0	0	0	72	0	0	0	3440	0	0	0	47	0	0	0	24
*Zacco platypus*	0	0	3	0	0	0	7	0	0	0	3	0	0	0	5	0	0	0	813	0	0	0	9	0

**TABLE A3:** Species composition in each sites based on sequence abundance of top 5.

Sampling sites	Species composition based on sequence abundance of top 5
MFT	*Carassius auratus* (13.41%) *Cyprinus carpio* (13.04%) *Ctenopharyngodon idellus* (9.49%) *Pelteobagrus fulvidraco* (8.55%) *Hemiculter tchangi* (8.29%)
QJK	*Carassius auratus* (40.22%) *Cyprinus carpio* (10.3%) *Ctenopharyngodon idellus* (8.05%) *Pelteobagrus fulvidraco* (6.26%) *Hemiculter tchangi* (5.87%)
DJT	*Cyprinus carpio* (18.59%) *Hemiculter tchangi* (14.77%) *Hypophthalmichthys nobilis* (9.54%) *Carassius auratus* (8.12%) *Pelteobagrus fulvidraco* (7.87%)
GZT	*Carassius auratus* (14.82%) *Cyprinus carpio* (12.5%) *Pelteobagrus fulvidraco* (7.82%) *Ctenopharyngodon idellus* (5.7%) *Hemiculter tchangi* (5.35%)
SJ	*Ctenopharyngodon idellus* (18.72%) *Cyprinus carpio* (18.25%) *Carassius auratus* (11.31%) *Oreochromis* sp. (10.44%) *Silurus asotus* (6.04%)
SPH	*Cyprinus carpio* (17.41%) *Carassius auratus* (13.13%) *Pelteobagrus fulvidraco* (8.22%) *Hemiculter tchangi* (7.14%) *Ctenopharyngodon idellus* (5.88%)
